# A lipocalin mediates unidirectional heme biomineralization in malaria parasites

**DOI:** 10.1073/pnas.2001153117

**Published:** 2020-06-29

**Authors:** Joachim M. Matz, Benjamin Drepper, Thorsten B. Blum, Eric van Genderen, Alana Burrell, Peer Martin, Thomas Stach, Lucy M. Collinson, Jan Pieter Abrahams, Kai Matuschewski, Michael J. Blackman

**Affiliations:** ^a^Malaria Biochemistry Laboratory, The Francis Crick Institute, NW1 1AT London, United Kingdom;; ^b^Department of Molecular Parasitology, Institute of Biology, Humboldt University, 10115 Berlin, Germany;; ^c^Laboratory of Nanoscale Biology, Division of Biology and Chemistry, Paul Scherrer Institute, 5232 Villigen, Switzerland;; ^d^Electron Microscopy Science Technology Platform, The Francis Crick Institute, NW1 1AT London, United Kingdom;; ^e^Center for Cellular Imaging and NanoAnalytics, Biozentrum, University of Basel, 4051 Basel, Switzerland;; ^f^Institute of Biology, Leiden University, 2311 EZ Leiden, The Netherlands;; ^g^Faculty of Infectious and Tropical Diseases, London School of Hygiene & Tropical Medicine, WC1E 7HT London, United Kingdom

**Keywords:** malaria, *Plasmodium*, lipocalin, PV5, hemozoin

## Abstract

During blood-stage development, malaria parasites are challenged with the detoxification of enormous amounts of heme released during the proteolytic catabolism of erythrocytic hemoglobin. They tackle this problem by sequestering heme into bioinert crystals known as hemozoin. The mechanisms underlying this biomineralization process remain enigmatic. Here, we demonstrate that both rodent and human malaria parasite species secrete and internalize a lipocalin-like protein, PV5, to control heme crystallization. Transcriptional deregulation of *PV5* in the rodent parasite *Plasmodium berghei* results in inordinate elongation of hemozoin crystals, while conditional *PV5* inactivation in the human malaria agent *Plasmodium falciparum* causes excessive multidirectional crystal branching. Although hemoglobin processing remains unaffected, PV5-deficient parasites generate less hemozoin. Electron diffraction analysis indicates that despite the distinct changes in crystal morphology, neither the crystalline order nor unit cell of hemozoin are affected by impaired PV5 function. Deregulation of *PV5* expression renders *P. berghei* hypersensitive to the antimalarial drugs artesunate, chloroquine, and atovaquone, resulting in accelerated parasite clearance following drug treatment in vivo. Together, our findings demonstrate the *Plasmodium*-tailored role of a lipocalin family member in hemozoin formation and underscore the heme biomineralization pathway as an attractive target for therapeutic exploitation.

## Significance

During blood-stage development, the malaria parasite replicates inside erythrocytes of the vertebrate host, where it engulfs and digests most of the available hemoglobin. This results in release of the oxygen-binding prosthetic group heme, which is highly toxic in its unbound form. The parasite crystallizes the heme into an insoluble pigment called hemozoin, a process that is vital for parasite survival and which is exploited in antimalarial therapy. We demonstrate that the parasite uses a protein called PV5 in hemozoin formation and that interfering with PV5 expression can increase the parasite’s sensitivity to antimalarial drugs during blood infection. An improved understanding of the mechanisms underlying heme sequestration will provide valuable insights for future drug development efforts.

The devastating pathology of malaria is caused by infection of red blood cells with unicellular *Plasmodium* parasites, which reside within an intraerythrocytic parasitophorous vacuole (PV) (1). Throughout blood-stage development, the parasite ingests and catabolizes up to 80% of the host cell cytoplasm, facilitating amino acid acquisition and making sufficient room for parasite growth (2, 3). Hemoglobin is incorporated through endocytosis and then degraded by an array of functionally redundant proteases, a process that occurs in acidified lysosome-like organelles with species-specific morphology (4). In the rodent-infective parasite species *Plasmodium berghei*, one or more food vacuoles (FVs) give rise to small digestive vesicles (DVs), which only fuse at the very end of intraerythrocytic development (5). In contrast, the most virulent agent of human malaria, *Plasmodium falciparum*, directs all endosomal traffic to a single large FV, where proteolysis occurs (6).

Hemoglobin digestion is accompanied by the release of high levels of the porphyrin cofactor heme from the globin chains. Heme can damage proteins and lipids through various mechanisms, including the formation of free radicals (7). The unique challenge of heme detoxification is met by the parasite’s capacity to sequester the released heme into a bioinert crystalline product called hemozoin (Hz), which accumulates in the FV or DVs. Heme is initially oxidized to yield hematin, which then dimerizes through the reciprocal coordination of iron and propionate moieties. This molecular unit then assembles into Hz crystals that typically take the form of triclinic high aspect ratio parallelograms (8–10). By the end of intraerythrocytic development, all of the Hz crystals are contained within a central residual body, which is eventually released upon parasite egress from the host cell and which contributes to the inflammatory responses associated with acute malaria (11). The mechanism of Hz formation is highly debated. While several studies suggest a physicochemical and autocatalytic crystallization process (12–15), there have been reports of parasite proteins (16–18) and lipids (19–21) promoting Hz assembly in vitro.

The parasite’s dependency on heme detoxification has long been exploited in antimalarial therapy with outstanding success. Aminoquinolines inhibit Hz formation via direct physical interactions with hematin and the crystal surface, eventually leading to the build-up of cytotoxic free heme (22–25). The aminoquinoline chloroquine was the front-line medication against malaria from the 1950s onward until the emergence of widespread drug resistance restricted its utility (26). Nonetheless, to this day chloroquine remains among the most effective antimalarial drugs ever developed, highlighting the outstanding importance of heme sequestration for *Plasmodium* survival. It is thus crucial for future drug development efforts to gain a better understanding of the mechanisms underlying this unique biomineralization event.

In this report, we demonstrate that a parasite-encoded lipocalin called PV5 is crucial for physiological heme biomineralization in *Plasmodium*.

## Results

### Malaria Parasites Encode a Lipocalin-Like Protein, PV5.

**Fig. 1.**
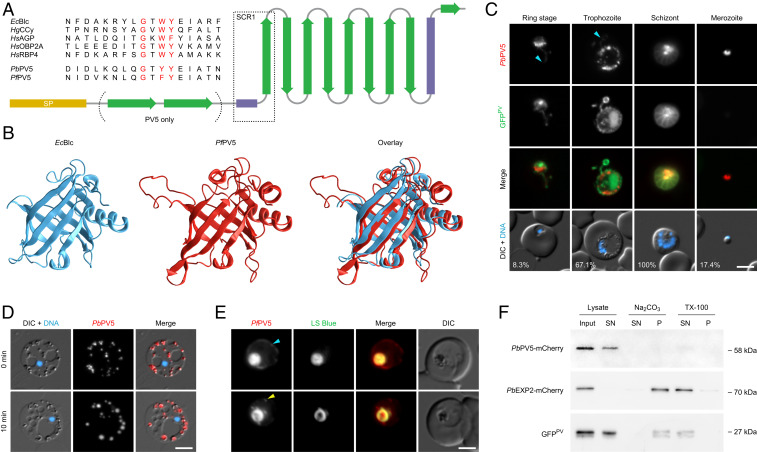
The *Plasmodium* lipocalin PV5 is trafficked to the parasite digestive compartments. (*A*) PV5 is a lipocalin family member. Secondary structure of *Plasmodium* PV5. Yellow, signal peptide (SP); green, β-strands; purple, helices. Note the two amino-terminal β-strands specific to PV5. Alignments of the SCR1 from different lipocalin family members and PV5 are shown in the upper left corner. Signature residues are highlighted in red. *Ec*Blc, *Escherichia coli* bacterial lipocalin; *Hg*CCy; *Homarus gammarus* (European lobster) crustacyanin; *Hs*AGP, *Homo sapiens* α_1_-acid glycoprotein; *Hs*OBP2A, *H. sapiens* odorant-binding protein 2A; *Hs*RBP4, *H. sapiens* retinol-binding protein 4; *Pb*/*Pf*PV5, PV5 from *P. berghei* and *P. falciparum*. (*B*) Structure homology modeling predicts a lipocalin fold for *Pf*PV5. Shown are the experimentally validated structure of *Ec*Blc (blue, *Left*, residues 27 to 175, PDB ID code 3MBT), the derived model of *Pf*PV5 (red, *Center*, residues 35 to 214) using *Ec*Blc as a homology template, and an overlay (*Right*). Modeling was performed with SWISS-MODEL and supported by I-TASSER. Amino acid sequence identity is 20%, similarity calculated from BLOSUM62 substitution matrix is 0.3. (*C*) Dual protein localization of PV5 to extensions of the PV and to intraparasitic structures in *P. berghei*. Transgenic parasites expressing the PV marker GFP^PV^ and the endogenous Pb*PV5* gene fused to mCherry-3xMyc were imaged live (27). Shown are the mCherry (red, first row) and GFP channels (green, second row), a merge of both signals (third row) and a merge of differential interference contrast images (DIC), and Hoechst 33342 nuclear stain (blue, fourth row). Cyan arrowheads, *Pb*PV5 in PV tubules. Numbers represent normalized mCherry intensity values obtained by quantitative live fluorescence microscopy. *n* = 44 parasites. (*D*) Intraparasitic PV5 localizes to Hz-containing DVs in *P. berghei*. Parasites (1 to 2 µL) were incubated under a coverslip (22 × 40 mm) for several minutes, leading to lysis of the host erythrocyte and the PV, and to mechanical expansion of the parasite (*Upper*). Shown are a merge of DIC and Hoechst 33342 nuclear stain (blue, first column), the signal of tagged *Pb*PV5 (red, second column), as well as a merge of all three channels (third column). Swelling of *Pb*PV5-containing DVs was observed 10 min later (*Lower*). Note the even distribution of *Pb*PV5 throughout the swollen DVs. (*E*) PV5 localizes to the central FV, intraparasitic vesicles and to the PV in *P. falciparum*. Transgenic parasites expressing the endogenous Pf*PV5* gene fused to mCherry were imaged live in the presence of Lysosensor blue DND-167 (LS Blue). Shown are the signals of mCherry (red, first column), LS Blue (green, second column), a merge of both signals (third column), and DIC images (fourth column). Cyan arrowhead, *Pf*PV5 in PV (*Upper*). Yellow arrowhead, *Pf*PV5 in small intraparasitic vesicles (*Lower*). (Scale bars, 5 µm.) (*F*) PV5 is a soluble protein. Subcellular fractionation was performed using the *Pb*PV5-tagged *P. berghei* line, which also expresses the soluble marker GFP^PV^, and a *P. berghei* line expressing the transmembrane protein *Pb*EXP2 fused to mCherry-3xMyc (62). Cell lysates were centrifuged and resulting membrane pellets were subjected to solubilization with Na_2_CO_3_ and Triton X-100 (TX-100). Input, supernatant (SN), and pellet fractions (P) were analyzed by Western blot using anti-mCherry and anti-GFP primary antibodies.

Employing a genome-wide in silico down-scaling approach, we previously identified an essential *P. berghei* PV protein, *Pb*PV5 (PBANKA_0826700), which has orthologs in all other *Plasmodium* species, including *P. falciparum* (*Pf*) (PF3D7_0925900) (27). Inspection of the PV5 amino acid sequence revealed a striking similarity to members of the functionally diverse lipocalin family, barrel-shaped proteins capable of binding various hydrophobic ligands and protein interaction partners (28). The signature lipocalin fold comprises a short amino-terminal 3_10_ helix followed by eight consecutive barrel-forming β-strands, an α-helix, and one more β-strand (Fig. 1*A*) (29). In addition, PV5 harbors two predicted preceding amino-terminal β-strands specific to *Plasmodium*, as well as a signal peptide. Multiple sequence alignments with lipocalins from phylogenetically distant organisms showed the presence of a highly conserved glycine and two aromatic residues within the structurally conserved region 1 (SCR1) of PV5, a hallmark of the extended calycin superfamily (Fig. 1*A*) (29). Among several structural homologs, the bacterial outer membrane lipocalin Blc from *Escherichia coli* was predicted to share the highest similarity with PV5. Homology modeling guided by the known *E. coli* Blc structure suggests that PV5 shares the overall architecture of the lipocalin family, including the characteristic β-barrel (Fig. 1*B*). Together, the sequence signatures and predicted structural features support membership of *Plasmodium* PV5 in the calycin protein superfamily.

### PV5 Is Trafficked to the Parasite Digestive Compartments.

We first investigated the spatiotemporal expression of *Pb*PV5 during asexual blood-stage development. Live fluorescence microscopy of transgenic *P. berghei* parasites expressing mCherry-3xMyc-tagged *Pb*PV5 confirmed that the protein localizes to tubular PV extensions during ring and trophozoite stages and surrounds individual daughter merozoites in segmented schizonts (Fig. 1*C*) (27). In addition, a substantial fraction of the protein was restricted to the parasite cytoplasm. This was particularly prominent in schizonts, where intraparasitic *Pb*PV5 appeared to localize to the Hz-containing residual body (Fig. 1*C*). In merozoites, mCherry fluorescence was concentrated in a punctate intraparasitic region, perhaps signifying storage of *Pb*PV5 in the dense granules, as has been demonstrated for several other important PV proteins (30–32), but this fraction was minimal as compared to the protein contained in the residual body. Quantification of the mCherry-fluorescence intensity in live parasites indicated that *Pb*PV5 is much more abundant in mature parasite stages than in rings and merozoites, suggesting substantial levels of de novo synthesis throughout parasite maturation (Fig. 1*C*). In trophozoites, the intraparasitic fraction of *Pb*PV5 was associated with spherical structures at the parasite periphery (Fig. 1*C*) and microscopic examination of mechanically expanded free parasites revealed that these were the Hz-containing DVs (Fig. 1*D*).

To test whether this localization is conserved across different *Plasmodium* species, we generated and imaged transgenic *P. falciparum* parasites expressing mCherry-tagged *Pf*PV5 (Fig. 1*E* and *SI Appendix*, Fig. S1). In younger parasite stages, fluorescence was restricted to one or more foci in the parasite periphery. With parasite maturation, most of the fluorescent signal overlapped with the Hz crystals and with the signal of the acidotropic dye Lysosensor blue DND-167, which accumulates in the acidic FV (Fig. 1*E* and *SI Appendix*, Fig. S1*D*). This is in good agreement with the reported detection of *Pf*PV5 in the proteome of purified *P. falciparum* FVs (33). In addition, we frequently observed staining of the PV and of small foci in the parasite cytoplasm (Fig. 1*E* and *SI Appendix*, Fig. S1*D*). Some trophozoites also showed staining of a perinuclear region corresponding to the endoplasmic reticulum (*SI Appendix*, Fig. S1*D*). As in *P. berghei*, faint fluorescent foci were observed associated with the budding merozoites in mature *P. falciparum* schizonts (*SI Appendix*, Fig. S1*D*).

Western blot analysis indicated the presence of full-length *Pf*PV5-mCherry in the transgenic *P. falciparum* parasites (*SI Appendix*, Fig. S1*C*). However, the predominant fraction of mCherry appeared to have been cleaved off *Pf*PV5, presumably by the FV-resident proteases. This cleavage was not observed in the tagged *P. berghei* line, possibly due to differences in the spacer regions linking *Pb*PV5 to the fluorescent protein. Subcellular fractionation and Western blot analysis revealed that full-length *Pb*PV5-mCherry is freely soluble (Fig. 1*F*). Together, our findings suggest that in both *Plasmodium* species, PV5 is trafficked to the PV via the secretory pathway and then internalized through endocytosis of host cell cytoplasm to eventually accumulate in the matrix of the parasite’s digestive compartments.

### Transcriptional Deregulation of Pb*PV5* Impairs Asexual Parasite Propagation In Vivo.

**Fig. 2.**
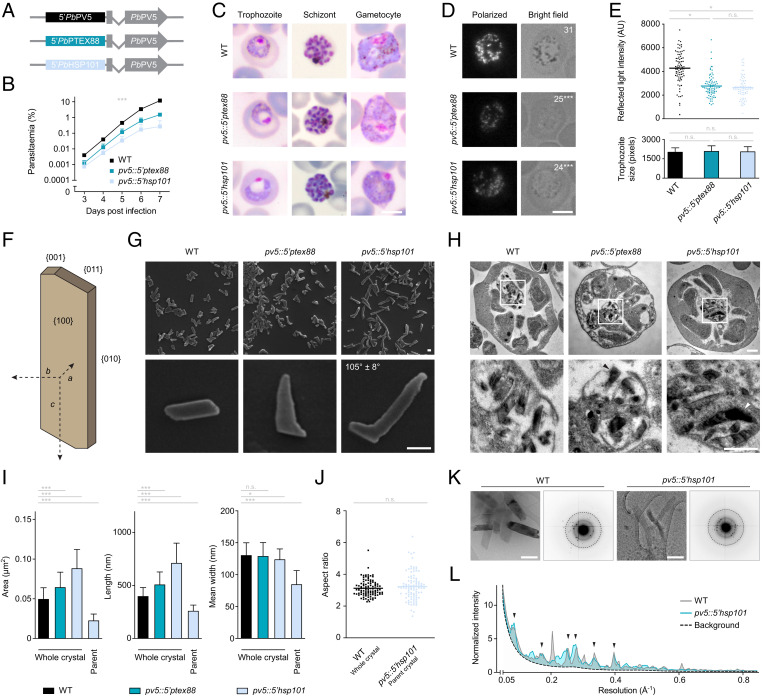
Deregulated expression of *PV5* impacts Hz formation in *P. berghei.* (*A*) Schematic representation of the genotypes of WT (*Top*), and transgenic *pv5::5′ptex88* (*Middle*) and *pv5::5′hsp101* parasites (*Bottom*). In the mutants, the endogenous Pb*PV5* promoter (black) was exchanged for the promoter of Pb*PTEX88* (dark blue) or Pb*HSP101* (light blue), respectively. (*B*) Reduced parasite proliferation upon *Pb*PV5 promoter swapping. Asexual blood-stage development was analyzed using the intravital competition assay (53). Average parasite multiplication rates were 11.4 (WT), 9.1 (*pv5::5′ptex88*), and 6.2 (*pv5::5′hsp101*). Shown are mean values ± SD. ***P < 0.001; two-way ANOVA. *n* = 3 independent infections. (*C*) Morphology of trophozoite, schizont, and gametocyte stages in the WT, *pv5::5′ptex88* and *pv5::5′hsp101* lines as observed by Giemsa staining. Note the lack of prominent dark pigment granules in mutant trophozoites and gametocytes as well as the dilation of the FV in mutant trophozoites. (Scale bar, 5 µm.) (*D* and *E*) Pb*PV5* is required for efficient Hz formation. (*D*) Trophozoites were visualized by polarization microscopy (*Left*) and bright field imaging (*Right*). Numbers indicate the mean quantity of bright puncta in polarization images. Significance values are shown for the comparison of the mutants with WT. (Scale bar, 5 µm.) (*E*) Quantitative polarization microscopy. Depicted are individual and mean intensity values of reflected polarized light in methanol-fixed WT, *pv5::5′ptex88* and *pv5::5′hsp101* trophozoites (bars, upper graph). Only trophozoites of similar size were analyzed (lower graph). Depicted are mean values ± SD. n.s., nonsignificant; *P < 0.05; ***P < 0.001; one-way ANOVA and Tukey’s multiple comparison test. *n* = 80 trophozoites from four independent infections. (*F*) Hz crystal architecture. In WT *Plasmodium* parasites Hz assembles as triclinic high aspect ratio parallelograms (64). Characteristic crystal axes and faces are indicated. (*G*) SEM images of Hz purified from WT, *pv5::5′ptex88* and *pv5::5′hsp101* mixed blood-stage parasites. The angle between the regularly shaped *pv5::5′hsp101* parent crystal and the outgrowth is indicated. *n* = 130 crystals. (Scale bars, 100 nm.) (*H*) Crystal morphology in situ. Shown are TEM images of WT, *pv5::5′ptex88* and *pv5::5′hsp101* schizonts (*Upper*) as well as their residual body at higher magnification (*Lower*). Abnormal crystal shapes in the mutants are indicated by arrowheads. (Scale bars, 500 nm.) (*I*) Hyperactive Hz growth is unidirectional. Shown are the dimensions of whole individual Hz crystals extracted from WT, *pv5::5′ptex88*, and *pv5::5′hsp101* parasites, including the area exposed to the electron beam (*Left*) as well as the length (*Center*) and mean width of the crystals (*Right*). The theoretical dimensions of the *pv5::5′hsp101* parent crystals were interpolated and are depicted as well. Shown are mean values ± SD. n.s., nonsignificant; *P < 0.05; ***P < 0.001; one-way ANOVA and Tukey’s multiple comparison test. *n* = 100 crystals. (*J*) Normal aspect ratio of *pv5::5′hsp101* parent crystals. Shown are the aspect ratios of whole Hz crystals from WT parasites and of the parent crystal from *pv5::5′hsp101*-generated Hz. Depicted are individual and mean values (bars). n.s., nonsignificant; Student’s *t* test. *n* = 100 crystals. (*K* and *L*) Unaltered crystalline order in Hz of *pv5::5′hsp101* parasites. (*K*) Depicted are TEM images (*Left*) of Hz purified from WT and *pv5::5′hsp101* parasites as well as their corresponding electron diffraction patterns (*Right*) showing comparable resolution of the Bragg peaks. Dashed circles demark a resolution of 0.5 Å^−1^. (Scale bars, 200 nm.) (*L*) Plot of the radial maximum diffracted intensity as a function of resolution. Data were normalized to the average median intensity at 0.18 Å^−1^ in order to correct for differences in diffracted volume. Arrowheads denote overlapping peaks. *n* = 10 (WT) and 18 (*pv5::5′hsp101*) diffraction datasets.

Our previous attempts to disrupt the genomic Pb*PV5* locus resulted only in atypical integration of the targeting construct without perturbing the endogenous gene, which is indicative of essential functions during the asexual blood-stage cycle in vivo (27). As an alternative genetic strategy to analyze Pb*PV5* function, we sought to deregulate Pb*PV5* expression by employing a promoter swap approach (Fig. 2*A*). Toward this aim, we generated parasites expressing the endogenous Pb*PV5* gene from the promoters of *Plasmodium* translocon of exported proteins 88 (*PTEX88*) or heat shock protein 101 (*HSP101*), respectively (*SI Appendix*, Fig. S2 *A* and *B*). Quantitative real-time PCR analysis of the mutants indicated that the knockdown efficiency was ∼60% in asynchronous blood stages (*SI Appendix*, Fig. S2*C*). Impaired growth prevented quantification of knockdown levels in synchronized ex vivo early blood stages. Strikingly, in the schizont stage, the mutants exhibited significantly elevated Pb*PV5* transcript levels, corresponding to ∼3.4 (*pv5::5′ptex88*) and ∼5.6-fold (*pv5::5′hsp101*) more than in WT schizonts (*SI Appendix*, Fig. S2*C*), showing that the promoter swap strategy was successful in deregulating the physiological expression of Pb*PV5* throughout the asexual replication cycle.

To investigate whether altered Pb*PV5* transcription results in reduced parasite fitness, we examined asexual propagation of the mutants in vivo. Growth of the promoter swap mutants was significantly impaired, with the *pv5::5′ptex88* parasites growing at 80% and *pv5::5′hsp101* parasites at only 54% of the WT growth rate (Fig. 2*B*). These results underscore the importance of correct Pb*PV5* expression during asexual replication of the parasite in vivo.

### Pb*PV5* Mutants Form Less Hz.

Inspection of Giemsa-stained thin blood films revealed striking morphological differences between WT parasites and the promoter swap mutants. In trophozoite stages, the FV of the mutants was significantly swollen, visible as a large bright area within the parasite cytoplasm close to the nucleus (Fig. 2*C*). Microscopic quantification revealed this area to be 1.5-fold (*pv5::5′ptex88*) or 1.8-fold (*pv5::5′hsp101*) larger than in WT parasites (*SI Appendix*, Fig. S3*A*). Comparison of the light intensities in the swollen FVs and the host cell cytoplasm provided no indication for an accumulation of native hemoglobin within the *Pb*PV5 mutants (*SI Appendix*, Fig. S3 *B* and *C*). The vacuolar swelling was transient, as mature schizonts did not exhibit comparable abnormalities (Fig. 2*C*).

Another striking phenomenon was the low visibility of dark granular material in mutant trophozoites, as compared to WT (Fig. 2*C*). This lack of granularity was most noticeable in the *pv5::5′hsp101* mutant and became particularly apparent during the gametocyte stages, where pigment granules are usually very prominent. To validate this finding, we subjected mixed blood-stage parasites to flow cytometry and measured the intensity of the side scattered light, a commonly used proxy for cellular granularity (*SI Appendix*, Fig. S3*D*). In agreement with our microscopic analysis, the Pb*PV5* mutants displayed reduced side scattering and the phenotype was again more severe in the *pv5::5′hsp101* mutant.

Because we suspected a Hz formation defect in the mutants, we fixed intraerythrocytic parasites with methanol and subjected them to polarization microscopy, which exploits the birefringent properties of Hz to specifically visualize the crystals. This approach revealed a weaker signal for the mutants, which correlated with reduced visibility of dark pigment in brightfield (Fig. 2*D*). Enumeration of individual bright entities showed the presence of fewer Hz-containing structures within the mutants (∼80% of WT) (Fig. 2*D*). Quantification of the polarized light intensity also indicated that *pv5::5′ptex88* parasites form only 64% and the *pv5::5′hsp101* 61% of the Hz generated in WT parasites (Fig. 2*E*). Together, these observations show that perturbation of Pb*PV5* expression results in reduced heme biomineralization in vivo.

### Protracted Hz Extension upon Deregulation of Pb*PV5* Expression.

We next aimed to examine how lower levels of Hz correlate with crystal size. To do this, we isolated Hz from mixed blood-stage parasites and examined the material by scanning electron microscopy (SEM). This confirmed the characteristic high aspect ratio parallelogram morphology of WT Hz (Fig. 2 *F* and *G*). Strikingly, this was not the case for crystals isolated from the promoter swap mutants. Hz from both transgenic parasite strains exhibited highly irregular shapes and rough edges and showed only few of the distinctive Hz crystal vertices (Fig. 2*G*). Crystals from *pv5::5′ptex88* parasites most often had a pointed and canine tooth-like appearance, extending from a single straight crystal face. These abnormalities were even more pronounced in the *pv5::5′hsp101* mutant, where in most cases there was a region of normal crystal morphology with two or three straight edges, corresponding to the {010}, {011}, and {001} faces. From this regular parent crystal emerged an enormous outgrowth that usually surpassed the dimensions of the parent crystal (Fig. 2*G*). This outgrowth consistently grew at an obtuse angle of ∼105° in relation to the dominant *c* axis of the parent crystal, although accurate determination of the angle was complicated by the slightly bent and irregular shape of the outgrowth, which might be attributed to the space restrictions encountered in the *P. berghei* DVs. The outgrowth’s angle was not reflected in the physiological morphology of Hz (Fig. 2 *F* and *G*) and at least two faces of the regular parent crystal appeared to be involved. Indeed, in most cases the outgrowth emerged from sites where the {010} and {011} faces meet and always grew along a plane corresponding to one of the original crystal faces (Fig. 2*G*). Other crystal formations were also observed, albeit at lower frequency, including some with multiple crystal branches and some with very rough surfaces (*SI Appendix*, Fig. S3*E*). We observed similar crystal abnormalities in situ by transmission electron microscopy (TEM) of purified schizonts (Fig. 2*H*).

Despite the lower overall levels of Hz formed and the abnormal crystal morphology, we found that the mutants formed larger Hz crystals, as indicated by the area exposed to the SEM electron beam (Fig. 2*I*). The *pv5::5′hsp101* mutant formed the largest crystals, which were ∼180% of WT size, while the *pv5::5′ptex88* crystals were at 130%. Importantly, the mutant Hz crystals displayed greater dimensions only in length but not in width because of the unidirectional expansion of the outgrowth (Fig. 2*I*). Examination of the parent crystals from *pv5::5′hsp101* parasites showed that these were roughly half the size of whole WT crystals (Fig. 2*I*). The aspect ratios of WT crystals and the *pv5::5′hsp101* parent crystals were identical, together indicating that a period of normal crystallization during early *pv5::5′hsp101* parasite development is followed by irregular crystal extension later on (Fig. 2*J*). Hz morphology was unaffected in an unrelated slow-growing mutant (34) and in chloroquine-treated WT parasites, suggesting that Hz crystal dysmorphism, as observed in the Pb*PV5* mutants, is not a phenomenon generally associated with poor parasite growth or mortality (*SI Appendix*, Fig. S3 *F* and *G*).

To determine whether the crystalline order was affected by deregulation of Pb*PV5*, we obtained electron diffraction patterns from WT- and *pv5::5′hsp101*-derived Hz (Fig. 2*K*) and analyzed the maximum diffracted intensities in concentric bins as a function of resolution. There was no difference in the drop-off of diffracted intensity between WT and mutant and the peak positions of the maxima corresponded (Fig. 2*L*). Differences in the magnitude of individual peaks can be attributed to preferential orientation, especially of the *pv5::5′hsp101*-derived crystals, which most often come to lie at their {100} faces. Together, these data indicate no differences in crystalline order or unit cell upon functional impairment of Pb*PV5.* We conclude that altered crystal morphology is not caused by alternative nucleation into a different hematin polymorph. Thus, deregulation of Pb*PV5* leads to the formation of ordered elongated Hz crystals with a highly variable and abnormal architecture.

### Loss of PV5 Causes Hz Branching in *P. falciparum*.

**Fig. 3.**
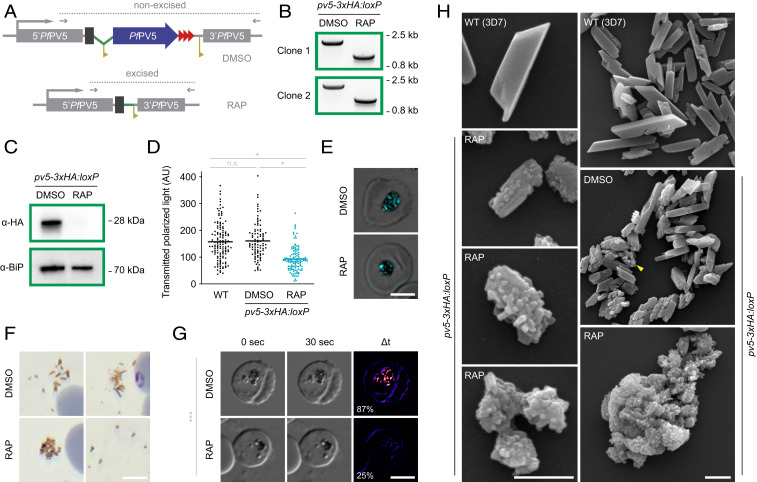
Absence of PV5 causes Hz branching in *P. falciparum*. (*A*) Schematic representation of DiCre-mediated Pf*PV5* disruption. The endogenous locus was modified to introduce *loxP* sites (yellow) flanking the majority of the coding sequence of the 3xHA (red)-tagged *Pf*PV5 (blue). The artificial intron is indicated in green. See also *SI Appendix*, Fig. S1*A*. Treatment with RAP induces Cre recombinase-mediated excision of the *loxP*-flanked sequence which results in truncation of Pf*PV5* leaving behind only the sequence encoding the protein’s signal peptide. Excision-sensitive primer combinations are indicated by arrows and expected diagnostic PCR fragments by dotted lines. (*B*) Diagnostic PCR of the modified Pf*PV5* locus following treatment with DMSO or RAP, respectively, using the primer combinations depicted in *A*. Results are shown from two independent *pv5-3xHA:loxP* clones. (*C*) Loss of *Pf*PV5 protein upon RAP treatment. Western blot analysis of parasite extracts following treatment with DMSO or RAP, respectively, using anti-HA and anti-*Pf*BiP primary antibodies. (*D* and *E*) *Pf*PV5 is required for efficient Hz formation. WT and *pv5-3xHA:loxP* parasites were treated with DMSO or RAP, respectively, and visualized by polarization microscopy 36 h after invasion. (*D*) Quantification of the polarized light intensity. Depicted are values from individual parasites as well as the mean intensity values (bars). n.s., nonsignificant; *P < 0.05; one-way ANOVA and Tukey’s multiple comparison test. *n* ≥ 93 parasites from three independent experiments. (*E*) Exemplary images of DMSO- and RAP-treated *pv5-3xHA:loxP* parasites. Shown is a merge of polarized light (cyan) and DIC. (Scale bar, 5 µm.) (*F*) Abnormal Hz morphology in the absence of *Pf*PV5. Hz released from residual bodies during parasite egress was imaged in Giemsa-stained thin culture smears of DMSO- and RAP-treated *pv5-3xHA:loxP* parasites. Note the spreading of elongated Hz crystals in DMSO-treated cultures and the clumping of granular Hz upon RAP treatment (*Left*). Only in very few instances did the crystals detach from one another in RAP-treated cultures (*Right*). (Scale bar, 5 µm.) (*G*) Reduced Hz movement in the FV upon loss of *Pf*PV5. Shown are DIC images of live DMSO- and RAP-treated *pv5-3xHA:loxP* parasites 36 h after invasion. Parasites were imaged twice at an interval of 30 s (*Left* and *Center*) and the difference of both images was visualized with pixel-by-pixel intensity subtraction (*Right*). Note the absence of Hz movement in the RAP-treated parasite (see also Movie S1). The percentage of parasites with moving Hz is indicated. ***P < 0.001; Student’s *t* test. *n* = 4 independent experiments with >300 parasites each. (Scale bar, 5 µm.) (*H*) SEM images of Hz purified from WT (3D7) and from DMSO or RAP-treated *pv5-3xHA:loxP* schizonts. (Scale bars, 500 nm.)

To investigate the consequences of *PV5* disruption, we generated a conditional *PV5* knockout line of the human pathogen *P. falciparum*, allowing rapamycin (RAP)-induced DiCre-mediated excision of the Pf*PV5* gene (Fig. 3*A* and *SI Appendix*, Figs. S1*A* and S4*A*). Correct modification of the locus was indicated by diagnostic PCR and by the successful tagging of *Pf*PV5 with a 3xHA tag, as demonstrated by Western blot and immunofluorescence analysis (Fig. 3 *B* and *C* and *SI Appendix*, Fig. S3 *B*–*E*). The 3xHA-tagged *Pf*PV5 localized to the PV and to intraparasitic vesicular structures (*SI Appendix*, Fig. S4*B*). Surprisingly, no signal was detected in the FV. However, global inhibition of the parasite’s cysteine proteases with E64 restored localization of 3xHA-tagged *Pf*PV5 to the FV and significantly increased the amount of tagged protein, together suggesting that the 3xHA tag is proteolytically cleaved upon FV delivery (*SI Appendix*, Fig. S4 *C* and *D*).

Treatment of *pv5-3xHA:loxP* parasites with RAP led to efficient gene excision and complete loss of *Pf*PV5 protein expression during the same intraerythrocytic cycle (Fig. 3 *B* and *C* and *SI Appendix*, Fig. S4*E*). This did not detectably affect parasite maturation but did result in a modest merozoite invasion defect upon rupture of the Pf*PV5*-null schizonts, reducing parasite replication (*SI Appendix*, Fig. S5). Extended monitoring of the RAP-treated parasites indicated an estimated fitness cost of ∼40% (*SI Appendix*, Fig. S5*B*). This is in good agreement with a proposed mutagenesis index score of 0.22 from a genome-wide *piggyBac* insertion screen (35). Accordingly, Pf*PV5*, although not essential under standard *P. falciparum* culture conditions, is required for optimal parasite propagation in vitro.

To examine the effects of Pf*PV5* ablation on heme biomineralization, Hz was visualized and quantified by polarization microscopy. RAP-treated *pv5-3xHA:loxP* parasites formed only 57% of the Hz observed in WT and DMSO-treated controls and individual crystals appeared to be globular rather than elongated (Fig. 3 *D* and *E*). In the absence of *Pf*PV5, Hz released at parasite egress no longer formed clusters of separate slender crystals but rather appeared as aggregates that only occasionally fell apart into individual units (Fig. 3*F*). In good agreement, microscopic inspection of live parasites revealed that the characteristic twirling motion of Hz within the central FV was lost upon Pf*PV5* knockout (Fig. 3*G* and Movie S1).

The dramatic abnormalities in Hz crystal morphology resulting from ablation of Pf*PV5* were even more evident by SEM analysis. While WT parasites formed crystals of the expected brick-like morphology, individual Hz units from RAP-treated *pv5-3xHA:loxP* parasites appeared smaller and more globular (Fig. 3*H* and *SI Appendix*, Table S1). The surfaces of these Hz units were covered in scales and stubby crystal buds. Individual crystals of comparable bud-like dimensions were not observed, indicating a branching rather than an aggregation phenomenon. In some instances, a lower number of crystal buds allowed the visualization of an ordered Hz core, suggesting that branching is initiated from a regular parent crystal (Fig. 3*H*). We detected several morphological intermediates between slightly scaled Hz, highly branched crystal units, and fused congregations (Fig. 3*H*). We frequently observed enormous aggregates of spherical proportions, mirroring the shape of the central FV (Fig. 3*H*). This suggested that hyperactive crystal branching in the absence of *Pf*PV5 caused individual studded Hz units to stick together and subsequently merge during Hz growth, which might explain the absence of motion in the parasite FV (Fig. 3*G*). NonRAP-treated *pv5-3xHA:loxP* control parasites mainly formed regular Hz crystals; however, 27.5% of the crystals exhibited a modest degree of branching (Fig. 3*H* and *SI Appendix*, Table S1). Furthermore, crystal size and aspect ratio were reduced in comparison to WT parasites, suggesting a moderate functional impairment of 3xHA-tagged *Pf*PV5 (Fig. 3*H* and *SI Appendix*, Table S1). Our data demonstrate that PV5 is critical for the efficient sequestration of heme and for the ordered expansion of Hz crystals in *P. falciparum*.

### Efficient Hemoglobin Processing in the Absence of *Pf*PV5.

**Fig. 4.**
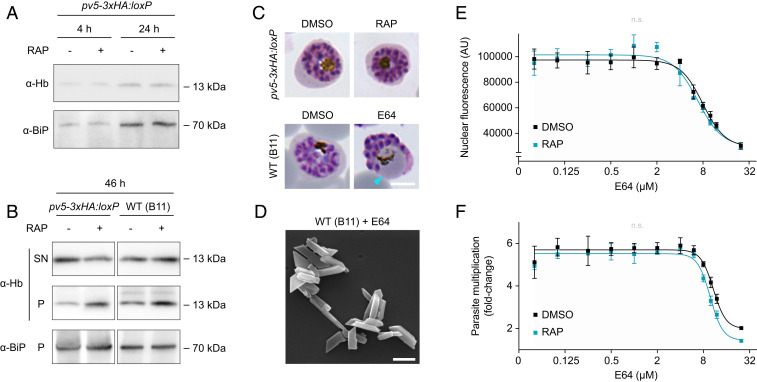
*Pf*PV5 regulates heme sequestration independently from hemoglobin processing. (*A* and *B*) Normal uptake and digestion of host cell hemoglobin by *Pf*PV5-deficient parasites. (*A*) Western blot analysis of induced and noninduced *pv5-3xHA:loxP* parasites released from their host cells by saponin lysis 4 and 24 h after invasion. (*B*) Forty-six hours after invasion, induced and noninduced *pv5-3xHA:loxP* or WT (B11) schizonts were released by saponin treatment. Resultant supernatants (SN) and schizont pellets were isolated. Blots were probed with antibodies directed against human hemoglobin α (Hb) and *Pf*BiP. Note an increase in intraparasitic hemoglobin upon RAP treatment in both *pv5-3xHA:loxP* and WT (B11) schizonts. (*C*) *Pf*PV5-deficient schizonts exhibit no vacuolar bloating. Shown are *pv5-3xHA:loxP* parasites treated from the ring stage onward with DMSO or RAP (*Upper*) and *P. falciparum* WT parasites treated from 24 h postinvasion onward with DMSO or 21.7 µM E64 (*Lower*). Cyan arrowhead, bloated food vacuole. (Scale bar, 5 µm.) (*D*) Inhibition of hemoglobin catabolism does not result in abnormal Hz morphology. Shown is an SEM image of Hz crystals isolated from the E64-treated *P. falciparum* WT parasites shown in *C*. (Scale bar, 500 nm.) (*E* and *F*) *Pf*PV5-deficient parasites display unaltered sensitivity toward E64. DMSO- and RAP-treated *pv5-3xHA:loxP* parasites were grown in various concentrations of E64 from the ring stage onward. (*E*) Nuclear SYBR Green fluorescence 44 h after invasion. (*F*) Parasite multiplication under static conditions following a 36-h incubation in the presence of E64 and subsequent inhibitor washout. Mean values ± SD are shown. n.s., nonsignificant; fitting of IC_50_ values following nonlinear regression, *n* = 3.

To exclude an indirect effect mediated by defective hemoglobin catabolism, we examined the hemoglobin content of saponin-released parasites. *Pf*PV5-deficient ring stages and trophozoites contained normal quantities of internalized hemoglobin (Fig. 4*A*). Only mature segmented *pv5-3xHA:loxP* schizonts exhibited slightly elevated concentrations of residual hemoglobin upon induction (Fig. 4*B*). However, this was also observed in WT schizonts upon RAP treatment, suggesting a minor compound-specific effect.

In WT parasites, inhibition of the vacuolar cysteine proteases with E64 caused significant bloating of the FV, because of an accumulation of undigested hemoglobin (Fig. 4*C*). A comparable bloating phenotype was not observed in untreated *Pf*PV5-null parasites, which retained normal FV morphology. We also noted that, unlike Pf*PV5* deletion, E64 treatment produced no changes in the architecture of Hz (Fig. 4*D*), and *Pf*PV5-deficient parasites retained normal E64 sensitivity (Fig. 4 *E* and *F*). Furthermore, saponin treatment released normal amounts of hemoglobin from schizont-infected erythrocytes in the absence of *Pf*PV5, indicating unaltered hemoglobin ingestion (Fig. 4*B*). Combined, these findings suggest that inhibition of hemoglobin catabolism does not directly translate into altered Hz morphology and that *Pf*PV5 is not involved in the overall consumption of host cell cytoplasm.

### Pb*PV5* Expression Influences Antimalarial Drug Sensitivity In Vivo.

**Fig. 5.**
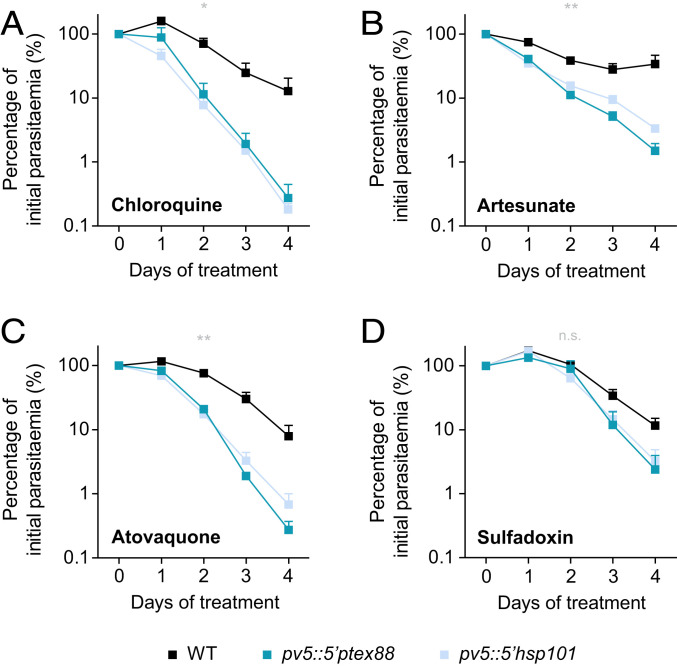
Targeting PV5 function results in parasite hypersensitivity toward antimalarial drugs in vivo. Enhanced drug susceptibility of the Pb*PV5* mutants in vivo. The 5 × 10^6^ mCherry-fluorescent *P. berghei* WT parasites were injected into SWISS mice together with 5 × 10^6^ GFP-fluorescent *pv5::5′ptex88* or *pv5::5′hsp101* parasites, respectively. From day 3 onward, mice were treated with curative doses of (*A*) chloroquine (288 mg/L in drinking water, ad libitum), (*B*) artesunate (50 mg/kg body weight, i.p.), (*C*) atovaquone (1.44 mg/kg body weight, i.p.) or (*D*) sulfadoxine (1.4 g/L in drinking water, ad libitum). Subsequent parasitaemia values were determined daily by flow cytometry of peripheral blood (41). Values are normalized in each case to the parasitaemia on day 0 of treatment. Shown are mean values ± SEM. n.s., nonsignificant; *P < 0.05; **P < 0.01; Two-way ANOVA. *n* = 3 independent experiments.

In the light of our evidence implicating PV5 in Hz formation, we next tested whether parasites with affected PV5 function display altered sensitivity toward chloroquine, a 4-aminoquinoline which is thought to inhibit heme biomineralization in *Plasmodium* (22–25). The absence of *Pf*PV5 did not detectably alter sensitivity toward chloroquine or any other tested antimalarial drug in cultured *P. falciparum* parasites (*SI Appendix*, Fig. S6). In stark contrast to this, however, the *P. berghei* promoter swap mutants responded to chloroquine treatment slightly earlier and disappeared from the circulation much more rapidly than WT parasites (Fig. 5*A*). A similar phenotype was observed upon treatment of infected mice with the artemisinin derivative artesunate, which has also previously been implicated in heme sequestration, although this remains contentious (36–40) (Fig. 5*B*). Most surprisingly, we also observed marked hypersensitivity of the *P. berghei* mutants toward atovaquone, a compound that targets the parasite’s mitochondrial electron transport chain (Fig. 5*C*), as well as a slight but nonsignificant increase in sensitivity toward the antifolate sulfadoxine (Fig. 5*D*). The relative survival levels of *pv5::5′ptex88* and *pv5::5′hsp101* on the fourth day of drug treatment suggested the greatest degree of hypersensitivity toward chloroquine (2.1% and 1.4% of WT survival, respectively), followed by atovaquone (3.5% and 8.6%) and artesunate (4.4% and 9.9%), and eventually sulfadoxine (20.5% and 27.8%). An unrelated slow-growing *P. berghei* mutant deficient in the maintenance of the mitochondrial membrane potential displayed normal sensitivity toward atovaquone using the same internally controlled assay (41), indicating that reduced parasite multiplication is unlikely to cause drug hypersensitivity. Collectively, these results suggest that interference with *PV5* expression critically enhances vulnerability of the parasite toward drug-mediated insult during in vivo blood infection.

## Discussion

In this work, we have demonstrated that Hz formation in malaria parasites involves a secreted calycin family member called PV5. While transcriptional deregulation of *PV5* in *P. berghei* resulted in protracted Hz elongation along a preexisting crystal plane, multidirectional branching was observed in the complete absence of PV5 in *P. falciparum*. Species differences aside, the disparity between those two phenotypes can be explained by the unique transcriptional dynamics observed in the *P. berghei* mutants. Here, the hyperactive crystal elongation that follows an initial phase of normal Hz growth coincides with a substantial increase in Pb*PV5* transcript abundance during late parasite development. In their physiological context, PTEX88 and HSP101 are coexpressed as members of the same protein complex, with HSP101 being more abundant than PTEX88 (42, 43), as supported by our qPCR analysis. Thus, the increased phenotypic severity in the *pv5::5′hsp101* mutant over the *pv5::5′ptex88* mutant indicates that the deficiencies in Hz formation can be attributed to late overexpression of PV5. Together with the chaotic Hz crystal branching observed in PV5-deficient *P. falciparum* parasites, this leads us to propose that PV5 acts as a facilitator of unidirectional Hz extension.

The highly polymorphic appearance of Hz in the absence of PV5 is reminiscent of the variable crystal morphology following β-hematin formation in vitro (12). While the exact biophysical mechanisms that govern PV5-mediated Hz morphogenesis remain to be delineated, our results support a model of conventional crystallization within the aqueous milieu of the parasite’s digestive compartments. The distinct changes in crystal morphology and branching behavior in the *PV5* mutants concur with established mineralogical phenomena and are difficult to reconcile with a lipid or protein-mediated polymerization scenario. It is interesting to speculate on the mechanisms by which PV5 may partake in heme biomineralization. In laboratory crystallization experiments, the extent of crystallographic mismatch branching is highly dependent on the degree of solute supersaturation, with high levels favoring the generation of novel crystal nuclei on the surface of the parent crystal (44). In contrast, lower degrees of supersaturation usually promote the expansion of preexisting crystals (44). It is conceivable that PV5 might reduce the extent of heme supersaturation by binding heme or hematin dimers, thereby moderating de novo Hz nucleation and promoting unidirectional crystal elongation. This could explain the Hz branching upon loss of PV5 in *P. falciparum*, as well as the prolonged crystal elongation and reduced Hz production upon late up-regulation of *PV5* in the *P. berghei* mutants. In support of this model, some lipocalin family members are known to specifically bind the heme degradation product biliverdin (45–47). Such interactions are unlikely to occur within the predicted PV5 β-barrel due to spatial constraints. However, although experimental validation for this is currently lacking, potential ligand binding sites might be located at the predicted prominently exposed loop between the barrel’s β-strands 5 and 6 (Fig. 1*B*) or at the extended amino terminus. The molecular functions of PV5 might also involve protein–protein interactions, as lipocalins from various organisms share the tendency to oligomerize and form complexes with different proteins and membrane receptors (28, 29).

The crystal branching characterizing PV5-deficient *P. falciparum* parasites could also be elicited by nonheme impurities adsorbing onto the crystal surface, where they would generate novel nucleation sites (44). In this alternative functional model, PV5 could act to bind these impurities to create a vacuolar environment permissive for proper biomineralization. *Pf*PV5-deficient parasites maintain an intact FV with a transvacuolar proton gradient as indicated by staining with Lysosensor blue DND-167 (*SI Appendix*, Fig. S7*A*). In the absence of indications for FV membrane damage, differences in leakage of impurities from the parasite cytoplasm appear unlikely. However, we cannot rule out the possibility that specific ions or organic compounds are more abundant in the vacuolar matrix of PV5-deficient parasites.

The striking localization pattern of PV5 is suggestive of initial secretion into the PV followed by endocytic uptake. Since we ruled out an involvement of PV5 in hemoglobin ingestion and catabolism, the transient vacuolar swelling in the *P. berghei* mutants likely reflects a secondary effect mediated by the grave deficiencies in heme sequestration. This is supported by a previous report demonstrating that defective hemoglobin catabolism causes chloroquine resistance in *P. berghei* (48), a phenotype that is in stark contrast to our own observations.

Our previous experiments had indicated that Pb*PV5* is essential for asexual blood-stage development in *P. berghei* (27), and the deregulation of Pb*PV5* transcription in the mutants described here indeed led to a striking fitness cost during in vivo infection. In contrast, complete ablation of Pf*PV5* in *P. falciparum* only resulted in a moderate fitness loss in vitro. The apparent dispensability of Pf*PV5* is therefore puzzling, but can be resolved by the notion that in vitro culture does not necessarily reflect all adversities encountered during host infection. For example, Hz-mediated stiffening of the residual body in the absence of PV5 might hinder the passage of infected cells through capillaries or through the interendothelial slits of the spleen, a scenario that would result in parasite elimination only during in vivo infection. Similarly, we observed enhanced drug susceptibility only in the *P. berghei* mutants. Thus, it appears plausible that any imbalance in heme biomineralization in the absence of PV5 is compensated for under optimal culture conditions, revealing its adverse effects only during in vivo infection. We thus propose that the molecular mechanism by which PV5 regulates Hz formation might involve host factors that are not encountered in a cell culture setting. The observation of enhanced drug susceptibility in the Pb*PV5* mutants is in agreement with the notion that aberrant heme biomineralization chemo-sensitizes parasites to partner drugs. Although oxidative stress and lipid peroxidation remained unchanged upon deletion of Pf*PV5* (*SI Appendix*, Fig. S7*B*), the reduced Hz formation efficiency in the *PV5* mutants suggests elevated heme concentrations within the parasite mediating the fitness loss and drug hypersensitivity.

In summary, we provide conclusive evidence for a parasite factor mediating Hz formation in vivo. Since this *Plasmodium*-encoded member of the calycin superfamily also governs parasite viability and susceptibility to drug-mediated insult during blood infection, our observations reinforce Hz formation as an excellent pathway for therapeutic intervention. Further investigation of the parasite heme detoxification machinery in vivo, as exemplified here for PV5, will significantly improve our understanding of this unique biomineralization process and holds great promise for the development of novel malaria intervention strategies.

## Materials & Methods

### Structure Homology Modeling.

Structure homology modeling was performed using the SWISS-MODEL server (49). *Pf*PV5 (residues 35 to 214) was aligned to the experimentally validated structure of *Ec*Blc (residues 27 to 175, PDB ID code 3MBT) (50), resulting in a global model quality estimate value of 0.39 and a qualitative model energy analysis value of −5.48. Modeling was confirmed with I-TASSER (51), which also identified *Ec*Blc (PDB ID code 2ACO) (52) as the most closely related structural analog of *Pf*PV5 with a template modeling score of 0.75. Due to a lack of sequence similarity, the structure of the extended *Pf*PV5 amino terminus was not modeled.

### *P. berghei* Cultivation.

*P. berghei* parasites were propagated in SWISS mice under constant drug pressure with pyrimethamine (70 mg/L in drinking water, ingested ad libitum; MP Biomedicals) to avoid homology-induced reversion of the promoter swap mutants to the original WT genotype. This was routinely checked by diagnostic PCR of genomic DNA as shown in *SI Appendix*, Fig. S2 *A* and *B*. Drug pressure was withdrawn 5 d prior to experimentation to avoid secondary effects of pyrimethamine treatment. Pyrimethamine-resistant Berred WT parasites (53) were treated accordingly. All infection experiments were carried out in strict accordance with the German “Tierschutzgesetz in der Fassung vom 22. Juli 2009” and the Directive 2010/63/EU of the European Parliament and Council “On the protection of animals used for scientific purposes.” The protocol was approved by the ethics committee of the Berlin state authority (“Landesamt fur Gesundheit und Soziales Berlin,” permit no. G0294/15).

*P. berghei* growth was determined with the previously described intravital competition assay (53). In short, 500 mCherry-fluorescent Berred WT and 500 GFP-fluorescent mutant blood-stage parasites were coinjected intravenously and parasitaemia was analyzed daily by flow cytometry. For drug sensitivity assays, 5 × 10^6^ WT and 5 × 10^6^ mutant parasites were coinjected intravenously. Drug treatment as well as daily flow cytometric parasite detection were commenced 3 d later (41). Mice were treated with curative doses of chloroquine (288 mg/L in drinking water, ingested ad libitum; Sigma Aldrich), atovaquone (1.44 mg/kg body weight per day injected intraperitoneally; GlaxoSmithKline), artesunate (50 mg/kg body weight per day injected intraperitoneally; Sigma Aldrich), or sulfadoxine (1.4 g/L in drinking water, ingested ad libitum; Sigma Aldrich). Mixed blood stages and schizonts were purified by Nycodenz gradient centrifugation (54).

### *P. falciparum* Cultivation.

*P. falciparum* parasites were propagated in type AB^+^ human red blood cells at 90% N_2_, 5% CO_2_, and 5% O_2_ at 37 °C in RPMI 1640 containing AlbuMAXII (Thermo Fisher Scientific) supplemented with 2 mM l-glutamine. Parasites were routinely synchronized using a combination of Percoll gradient centrifugation and sorbitol lysis and were treated with 100 nM RAP, various concentrations of E64 or equivalent volumes of DMSO from the early ring stage onward. Growth assays were performed as described previously (55) and parasitaemia as well as DNA content were measured by flow cytometry using the nuclear dye SYBR Green (1:10,000; Thermo Fisher Scientific). For invasion assays, mature schizonts were incubated at 2% initial parasitaemia under static or shaking (120 rpm) conditions, as confirmed by flow cytometry. Parasitaemia was measured again 24 h after inoculation and the fold-change was calculated. For drug- and inhibitor-response analyses, *pv5-3xHA:loxP* ring-stage cultures at 0.5 to 2% parasitaemia were treated with varying concentrations of chloroquine, WR99210, artesunate, atovaquone, or E64 in the presence of DMSO or RAP in a 96-well format. Parasitaemia was determined 3 d later and the fold-change was calculated. E64 was washed out after 36 h to allow for reinvasion. In addition, nuclear SYBR Green fluorescence was determined by flow cytometry following 44 h of continuous E64 treatment.

### Generation and Validation of Transgenic *Plasmodium* Parasites.

To generate the Pb*PV5* promoter swap mutants, the 5′ portion of the Pb*PV5* gene (PBANKA_0826700) was PCR-amplified and cloned into the B3D^+^ vector using BamHI and SacII. The promoters of Pb*PTEX88* (PBANKA_0941300) or Pb*HSP101* (PBANKA_0931200) were then amplified and cloned in front of the start codon using BamHI (*SI Appendix*, Fig. S2*A*). Vectors were linearized with BstBI and transfected into GFP-fluorescent *P. berghei* Bergreen WT parasites, using standard protocols (54, 56, 57). Transgenic parasites were selected for with pyrimethamine and isolated by limiting dilution cloning. Pb*PV5* transcript abundance in the WT and the promoter swap mutants was measured by quantitative real-time PCR (qPCR) and normalized to *Pb*18S rRNA.

The conditional Pf*PV5* knockout lines were generated using established Cas9-mediated techniques. In short, *P. falciparum* B11 parasites constitutively expressing DiCre were cotransfected with a pDC2 guide plasmid inducing Cas9-mediated double strand cleavage of the Pf*PV5* locus (PF3D7_0925900), together with a linearized repair template (58–60) (*SI Appendix*, Fig. S3*A*). The template was generated by gene synthesis and contained 5′ and 3′ homology arms and a recodonised 3xHA-tagged Pf*PV5* sequence. The endogenous intron of Pf*PV5* was replaced with an artificial intron containing a *loxP* site (59). A second *loxP* sequence was introduced downstream of the Pf*PV5* stop codon. For live imaging of *Pf*PV5, mCherry was cloned into the conditional knockout vector in frame with the recodonised Pf*PV5* sequence using AatII. Transgenic parasites were selected with WR99210 and cloned by limiting dilution using of a previously described plaque assay (61). Primers used for molecular cloning, diagnostic PCR, and qPCR are indicated in Fig. 3*A* and in *SI Appendix*, Figs. S1*A* and S2*A* and Table S2.

### Fluorescence Microscopy.

The transgenic *P. berghei* parasite line *pv5-tag-GFP*^PV^ (27) was imaged live using an AxioImager Z2 epifluorescence microscope equipped with an AxioCam MR3 camera (Zeiss). For mechanical parasite expansion, 1 to 2 µL of infected blood was incubated under a 22 × 40-mm coverslip for several minutes until erythrocyte lysis became apparent. *P. falciparum* parasites were imaged on an Eclipse Ni light microscope (Nikon) fitted with a C11440 digital camera (Hamamatsu). Immunofluorescence analysis was performed with *P. falciparum pv5-3xHA:loxP* parasites that were fixed in 4% formaldehyde using rat anti-HA (1:500; Sigma Aldrich) and rabbit anti-SERA5 (1:500) (55) primary antibodies in combination with appropriate fluorophore-coupled secondary antibodies (1:1,000; Thermo Fisher Scientific). Staining with Lysosensor blue DND-167 (Thermo Fisher Scientific), BODIPY 581/591 C11 (Image-iT Lipid Peroxidation Kit, Thermo Fisher Scientific), and CellROX Green (Thermo Fisher Scientific) was performed according to the manufacturer’s instructions.

### Scanning Electron Microscopy.

Hz was isolated from Nycodenz (*P. berghei*) or Percoll (*P. falciparum*) enriched infected red blood cells. Cells were lysed in water at room temperature for 20 min, followed by a 10-min centrifugation step at 17,000 × *g*. The pellet was resuspended in 2% SDS in water and centrifuged as above. Three more washing steps with 2% SDS were then followed by three washing steps with distilled water, before the crystals were resuspended and transferred onto round glass coverslips (12 mm), where they were dried. Coverslips were mounted on SEM specimen stubs, sputter-coated, and then imaged on a LEO 1430 (Zeiss) or on a Quanta FEG 250 SEM (Thermo Fisher Scientific).

### Transmission Electron Microscopy.

Infected erythrocytes were fixed in 2.5% glutaraldehyde, embedded in beads of 2% agarose, treated with 1% osmium tetroxide, and further contrasted *en bloc* using 0.5% uranyl acetate. Following dehydration in a graded series of ethanol and propylene oxide, beads were embedded in epoxy resin and cured at 60 °C for at least 24 h. The 60-nm sections were produced with a Reichert Ultracut S ultramicrotome (Leica) using a diamond knife. Sections were retrieved on copper hexagonal mesh grids, and stained with 2% uranyl acetate and Reynold’s lead citrate before imaging on an EM 900 TEM (Zeiss) equipped with a wide-angle slow-scan 2K CCD camera (Tröndle Restlichtverstärkersysteme).

### Electron Diffraction.

Purified Hz was added to glow-discharged Lacey carbon films on 400-mesh copper grids, which were then transferred to a Vitrobot Mark IV plunge freezer (Thermo Fisher Scientific) with 100% humidity at 7 °C. The grids were blotted for 3 s with blot force 1 and plunged frozen in liquid ethane cooled by liquid nitrogen. Electron diffraction data were collected on a Talos cryoelectron microscope (Thermo Fisher Scientific) operated at 200 keV equipped with a hybrid pixel Timepix detector (512 × 512 pixels, 55 × 55 µm pixel size; Amsterdam Scientific Instruments). Still and rotation (70°) datasets were collected with a beam size of 2 µm. The recording time varied between 15 and 100 s. To determine the resolution, a powder pattern of an aluminum diffraction standard was recorded. Since the Hz crystals had a strong tendency to stick together, we measured diffraction data of crystal clusters. For each individual dataset, we determined the location of the central electron beam and shifted the patterns to make the beams coincide. Since crystal clustering prevented indexing of the diffraction data, the radial median and maximum intensities were determined as a function of resolution. Hereafter, the WT and *pv5::5′hsp101* datasets were averaged and normalized to the local background median intensity at 0.18 Å^−1^.

### Subcellular Fractionation and Immunoblotting.

*P. falciparum* parasites were released from erythrocytes by treatment with 0.15% saponin in PBS. Murine erythrocytes infected with the *pv5-tag-GFP*^PV^ or *exp2-mCherry P. berghei* lines (62) were purified on a Nycodenz gradient and lysed hypotonically for 1 h on ice in 10 mM Tris⋅HCl, pH 7.5. *P. berghei* lysates were centrifuged for 50 min at 100,000 × *g*. Membrane pellets were resuspended in 0.1 M Na_2_CO_3_ in PBS or in 1% Triton X-100 in PBS, respectively, and centrifuged for 50 min at 100,000 × *g*. Proteins were separated on SDS-polyacrylamide and transferred to nitrocellulose membranes. Western blotting was performed using rat anti-mCherry (1:5,000; ChromoTek), chicken anti-GFP (1:5,000; Abcam), rat anti-HA (1:1,000; Sigma Aldrich), rat anti-*Pf*BiP (1:1,000) (63), and rabbit anti-human hemoglobin α-primary antibodies (1:1,000; Abcam) followed by chemiluminescence detection with horseradish peroxidase-coupled secondary antibodies (1:10,000; Sigma Aldrich, or 1:5,000; Jackson ImmunoResearch).

### Quantitative Hz Analysis.

Hz was visualized and quantified microscopically in methanol-fixed infected red blood cells. Hz from *P. berghei* was analyzed by reflection contrast polarized light microscopy using a Leica DMR widefield microscope equipped with a ProgRes MF camera (Jenoptik) and the POL filter set 513813 (Leica). Hz from *P. falciparum* was analyzed by transmitted polarized light (488 nm) microscopy using an LSM 710 confocal microscope (Zeiss) equipped with a crossed polarizer in the condenser. Cellular granularity was approximated by quantification of side scattered light using an LSRFortessa flow cytometer (BD Biosciences). Hz crystal dimensions were analyzed using FIJI. Due to the variation in the *P. berghei* mutant’s crystal width, individual crystals were divided into nine evenly spaced segments along the dominant axis. The width of each segment was determined and the values were averaged. For the *pv5::5′hsp101* parent crystal and the crystal outgrowth, transects were drawn through the central axis of either structure and their shared angle was determined. Hz movement within the FV of *pv5-3xHA:loxP* parasites was imaged live 36 h following treatment with DMSO or RAP.

### Data Availability.

All data are presented in the main text or are available in the *SI Appendix*. Generated plasmids and parasite lines are available from the corresponding author.

## Supplementary Material

Supplementary File

Supplementary File
