# Supplementary material for: A lipocalin mediates unidirectional heme biomineralization in malaria parasites
Source: Proc Natl Acad Sci U S A. 2020 Jun 29;117(28):16546–56. doi: 10.1073/pnas.2001153117 (PMC7368307; doi:10.1073/pnas.2001153117)
Supplement: Supplementary File [file pnas.2001153117.sapp.pdf]

SI Appendix

A lipocalin mediates unidirectional haem biomineralization in malaria parasites

Joachim M. Matz, Benjamin Drepper, Thorsten B. Blum, Eric van Genderen, Alana Burrell, Peer Martin, Thomas Stach, Lucy Collinson, Jan Pieter Abrahams, Kai Matuschewski & Michael J. Blackman

Figure S1 Endogenous tagging with mCherry reveals delivery of PV5 to the *Plasmodium falciparum* food vacuole

Figure S2 Generation and validation of Pb*PV5* promoter swap mutants

Figure S3 Vacuolar dilation and defective haemozoin formation in the Pb*PV5* mutants

Figure S4 Localization and rapamycin-induced loss of 3xHA-tagged *Pf*PV5

Figure S5 Impaired fitness of *in vitro* cultivated *Plasmodium falciparum* in the absence of PV5

Figure S6 Normal drug sensitivity of PV5-deficient *P. falciparum* parasites *in vitro*

Figure S7 Absence of *Pf*PV5 does not cause dissipation of the vacuolar pH gradient nor an increase in oxidative stress

Movie S1 Absence of *Pf*PV5 ablates haemozoin movement within the food vacuole of *Plasmodium falciparum*. (Movie legend)

Table S1 Haemozoin crystal morphometry

Table S2 Primer sequences

www.pnas.org/cgi/doi/10.1073/pnas.2001153117

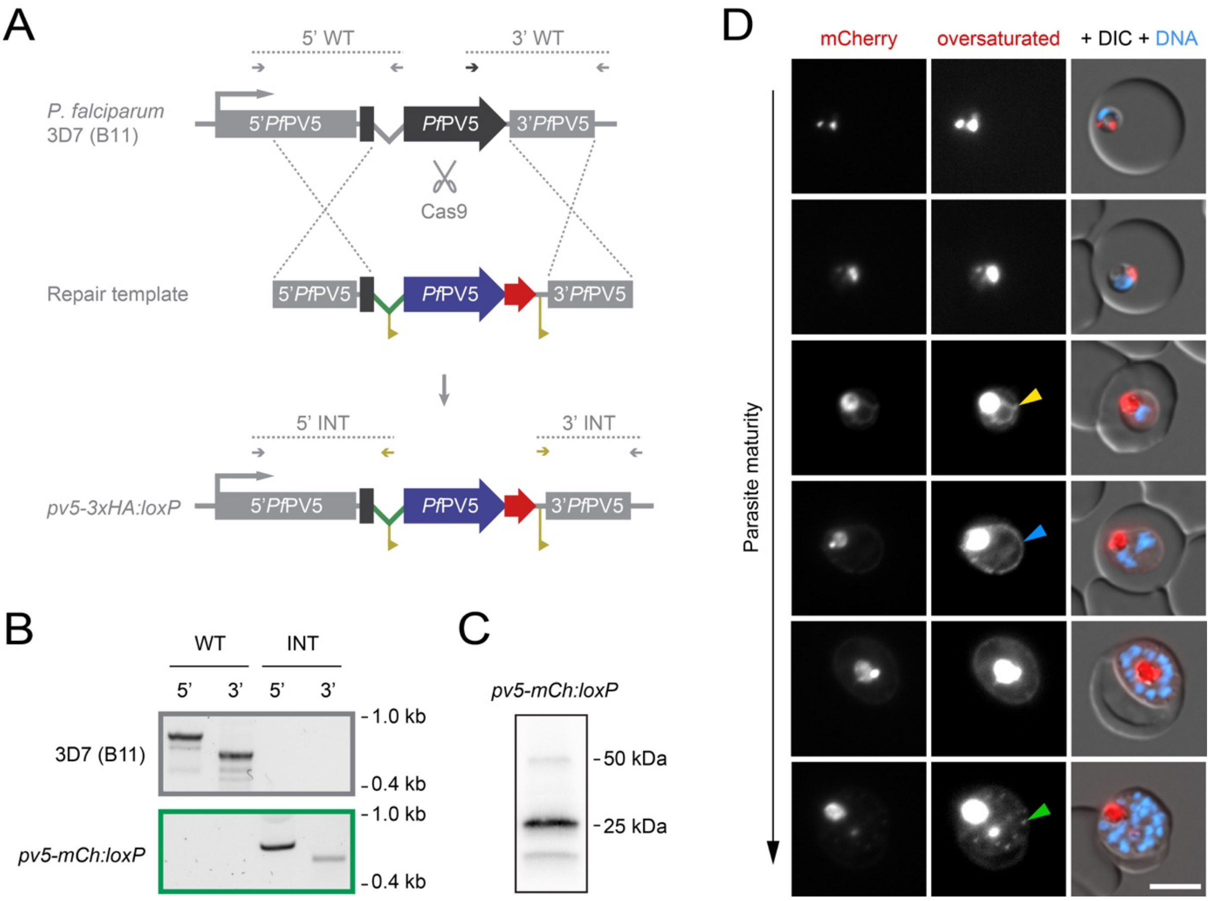

***SI Appendix*, Fig. S1.** Endogenous tagging with mCherry reveals delivery of PV5 to the *Plasmodium falciparum* food vacuole.

(*A*) Genetic strategy for tagging and conditional disruption of Pf*PV5*. The Pf*PV5* genomic locus (dark grey) was targeted by Cas9-mediated double strand cleavage and repaired by homologous recombination with a synthetic template containing 5' and 3' homology arms (light grey), a re-codonised version of Pf*PV5* (blue) fused to an mCherry or 3xHA tag (red) and *loxP* sequences (yellow) within the artificial intron (green) and following the stop codon. Wild-type (WT) and integration-specific primer combinations (INT) for diagnostic PCR are indicated by arrows and expected amplicons by dotted lines.

(*B*) Diagnostic PCR of the recipient *P. falciparum* 3D7 (B11) line (top) and of the isolated *pv5-mCh:loxP* clonal line (bottom) using the primer combinations depicted in *A*.

(*C*) Western blot analysis of non-induced *pv5-mCh:loxP* parasites using anti-mCherry primary antibodies. Signals corresponding to mCherry-tagged *Pf*PV5 (~50 kDa) and mCherry alone (~25 kDa) are observed.

(*D*) Live fluorescence imaging of non-induced *pv5-mCh:loxP* parasites throughout the intraerythrocytic life cycle. Shown are the fluorescent mCherry signal (red, left column) and a

merge with differential interference contrast images (DIC) and Hoechst 33342 nuclear stain (DNA, blue, right column). To enhance visibility of mCherry in the PV (blue arrowhead), in a perinuclear region most likely corresponding to the endoplasmic reticulum (yellow arrowhead), and in punctate structures within segmenting schizonts (green arrowhead), the mCherry channel has been oversaturated (middle column). Scale bar, 5 µm.

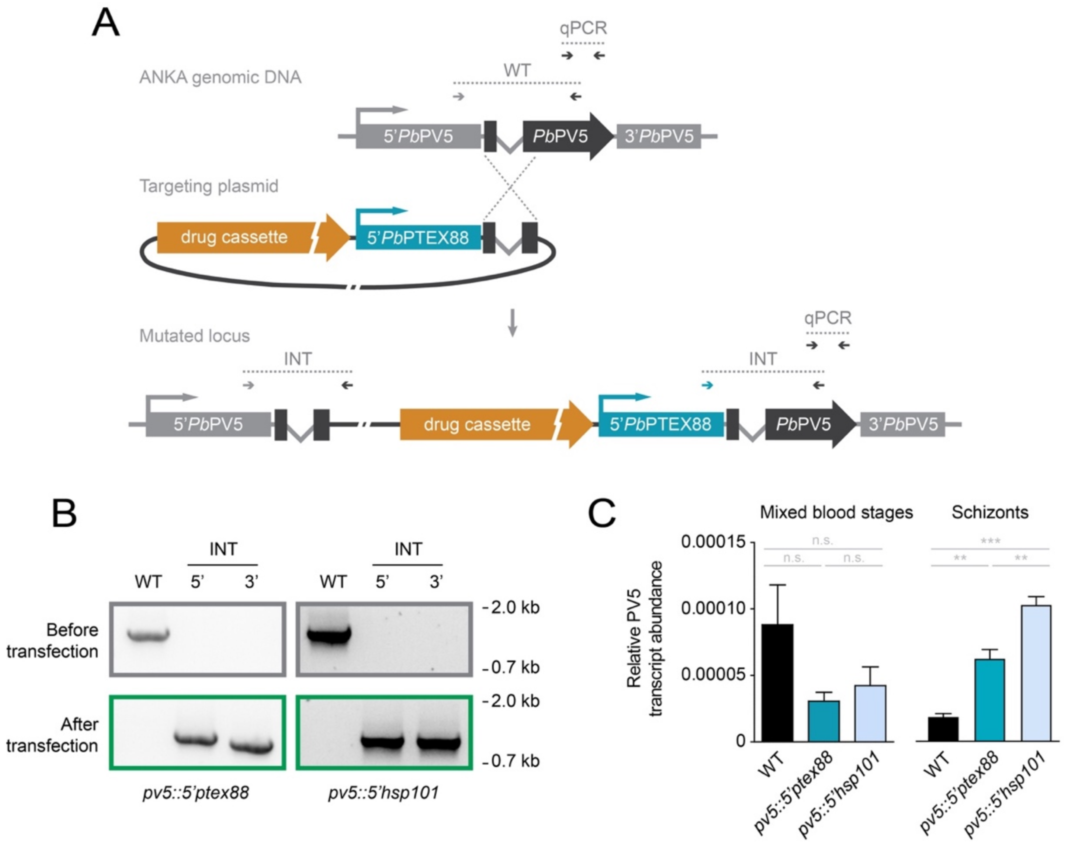

***SI Appendix*, Fig. S2.** Generation and validation of Pb*PV5* promoter swap mutants.

(*A*) Genetic strategy to exchange the endogenous promoter (light grey) of Pb*PV5* (dark grey) using single homologous recombination. The endogenous Pb*PV5* locus was targeted with an insertion plasmid containing the promoter sequence of Pb*PTEX88* (shown, blue) or Pb*HSP101* (not shown) fused to the amino-terminal sequence of Pb*PV5* as well as the drug-selectable hDHFR-yFcu cassette (orange). Successful insertion yields parasites expressing full length Pb*PV5* from a heterologous promoter and a non-functional amino-terminal fragment from the endogenous promoter, encoding the *Pb*PV5 signal peptide. Primer combinations for wild-type (WT) and integration-specific reactions (INT) as well as for quantitative real-time PCR (qPCR) are indicated by arrows and expected amplicons by dotted lines.

(*B*) Diagnostic PCR of the WT locus (top) and of the drug-selected and isolated Pb*PV5* mutants (bottom) using the primer combinations depicted in *A*.

(*C*) Dynamic changes in Pb*PV5* transcription upon promoter swap. Mixed blood stages and mature segmented schizonts were purified from mouse blood or from *in vitro* culture, respectively, and subjected to qPCR using primers targeting a 3' segment of Pb*PV5*, as depicted in *A*. Pb*PV5* transcript abundance was normalized to *Pb*18S rRNA. Shown are mean values +/- SEM. n.s., non-significant; **, P<0.01; ***, P<0.001; One-way ANOVA and Tukey's multiple comparison test. N=6 for mixed blood stages and 3 for schizonts.

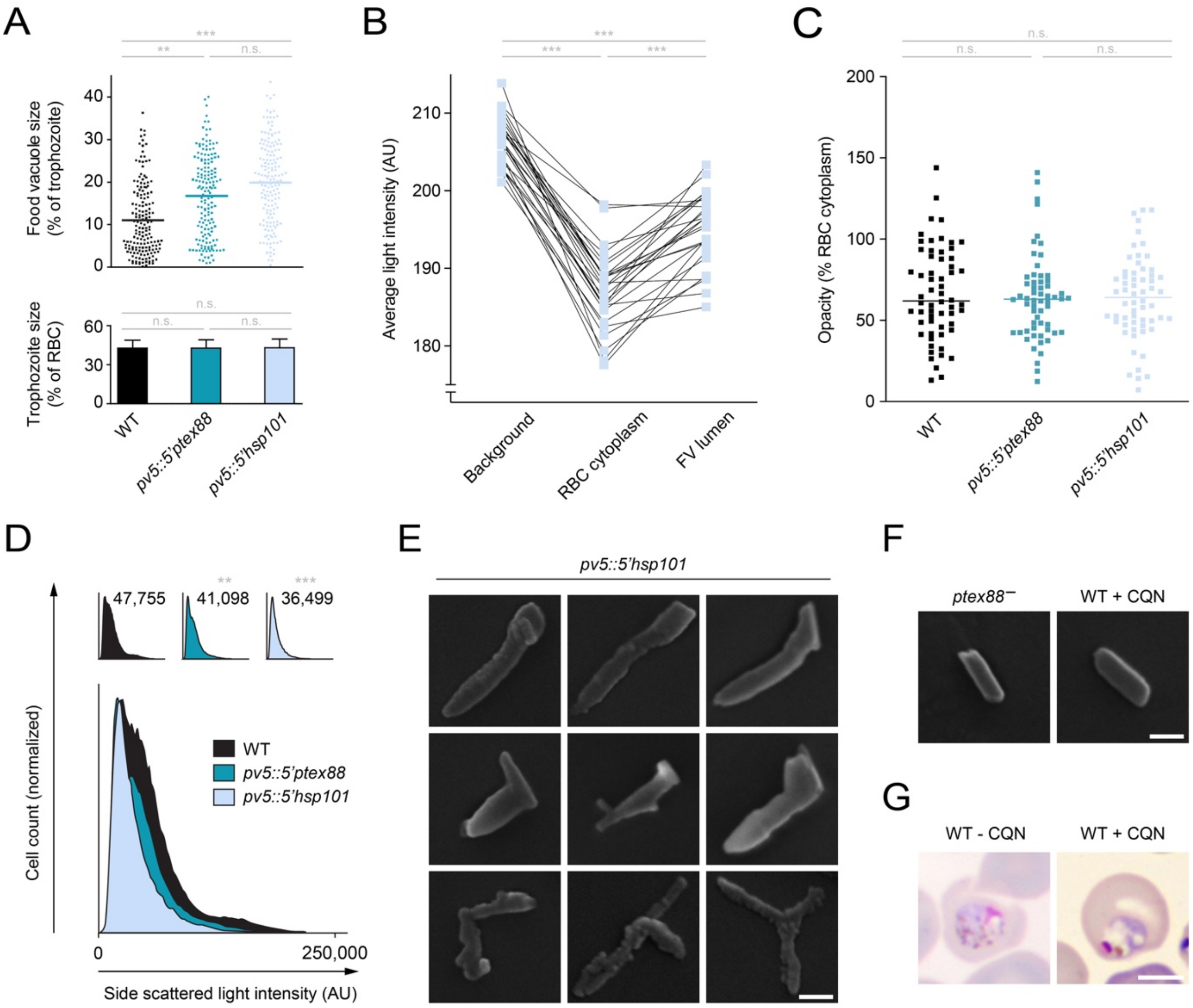

***SI Appendix*, Fig. S3.** Vacuolar dilation and defective haemozoin formation in the Pb*PV5* mutants.

(*A*) Quantification of vacuolar dilation. The translucent area observed in Giemsa-stained trophozoites corresponding to the FV was measured microscopically and expressed as the percentage of the entire trophozoite area (upper graph). Depicted are individual and mean values (bars). Only trophozoites of identical size were analysed (lower graph). Shown are mean values +/- SD. n.s., non-significant; **, P<0.01; ***, P<0.001; One-way ANOVA and Tukey's multiple comparison test. N=165 trophozoites from 5 independent infections.

(*B*) Vacuolar translucence in *pv5::5'hsp101* trophozoites. The light intensities of the *pv5::5'hsp101* FV, the erythrocyte cytoplasm and the background of Giemsa-stained blood smear were determined microscopically. Lines connect values from individual cells. ***, P<0.001; Repeated measures one-way ANOVA and Tukey's multiple comparison test. N=30 trophozoites from 2 independent infections.

(*C*) Normal opacity in the FVs of Pb*PV5* promoter swap mutants. Shown are inverted light intensity values of the FVs from WT and Pb*PV5* mutants relative to the erythrocyte cytoplasm

obtained by light microscopy of Giemsa-stained blood smears. n.s., non-significant; One-way ANOVA and Tukey's multiple comparison test. N=60 trophozoites from 2 independent infections.

(*D*) Pb*PV5* promoter swap mutants are less granular. Infected blood was subjected to flow cytometry and the intensity of the side scattered light was determined. Shown are individual histograms including the mean side scatter intensity values (top) as well as a merge of WT, *pv5::5'ptex88* and *pv5::5'hsp101* histograms (bottom). Significance values are shown for the comparison of the mutants with WT. n.s., non-significant; **, P<0.01; ***, P<0.001; One-way ANOVA and Tukey's multiple comparison test. N=6 independent infections.

(*E*) A selection of Hz crystals generated by *pv5::5'hsp101* parasites as visualized by scanning electron microscopy. Scale bar, 100 nm.

(*F*, *G*) Hz morphology is not affected by slow parasite growth or mortality. (*F*) Crystals were extracted from slow-growing *PTEX88* knockout parasites (left) and from WT parasites treated with curative doses of chloroquine (CQN, 288 mg/l in drinking water, *ad libitum*) (right) and were visualized by scanning electron microscopy. Scale bar, 100 nm. (*G*) Morphology of untreated (left) and CQN-treated WT parasites (right) as shown by Giemsa staining. Note the pigment clumping and vesiculation in the dying parasite. Scale bar, 5 µm.

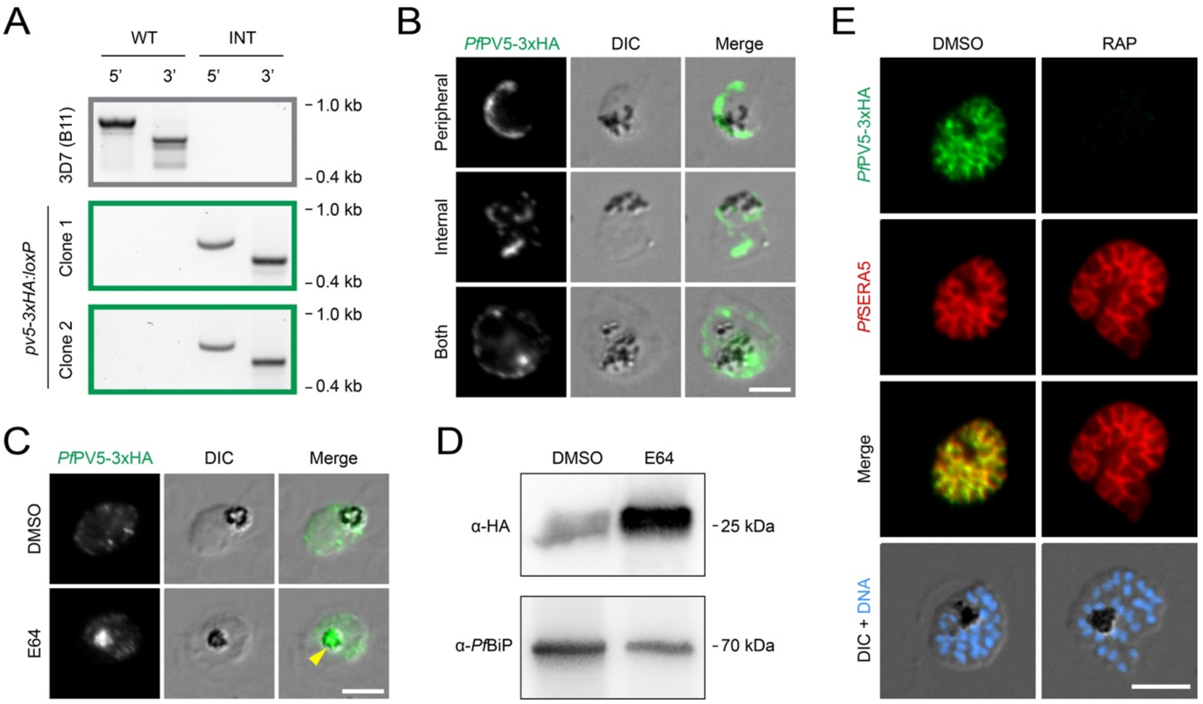

***SI Appendix*, Fig. S4.** Localization and rapamycin-induced loss of 3xHA-tagged *Pf*PV5.

(*A*) Diagnostic PCR of the recipient *P. falciparum* B11 line (top) and of two isolated *pv5-3xHA:loxP* clones (bottom) using the primer combinations depicted in *SI Appendix*, Fig. S1*A*.

(*B*) Localization of 3xHA-tagged *Pf*PV5. Immunofluorescence analysis was performed using anti-HA primary antibodies. Depicted are exemplary *pv5-3xHA:loxP* trophozoites and immature schizonts demonstrating localization of 3xHA-tagged *Pf*PV5 to the parasitophorous vacuole (PV, top), to internal parasite structures (centre) or both (bottom). Shown are the signal of tagged *Pf*PV5 (green, left), differential interference contrast images (DIC, middle) and a merge (right).

(*C*, *D*) Inhibition of FV cysteine proteases restores localization of 3xHA-tagged *Pf*PV5 to the FV. *pv5-3xHA:loxP* parasites were treated with dimethyl sulfoxide (DMSO) or 21.7 µM E64 from 24 hours after invasion onwards and parasites were examined 20 hours later by immuno-fluorescence (*C*) and Western blot analysis (*D*), using antibodies against HA and *Pf*BiP as a loading control. Yellow arrowhead, 3xHA-tagged *Pf*PV5 overlapping with Hz.

(*E*) Loss of *Pf*PV5 protein upon rapamycin (RAP) treatment. Immunofluorescence analysis of *pv5-3xHA:loxP* schizonts was performed with primary antibodies directed against HA and the PV protein *Pf*SERA5. Shown are the individual signals of 3xHA-tagged *Pf*PV5 (green, first row) and *Pf*SERA5 (red, second row), a merge of both signals (third row) as well as a merge of DIC with Hoechst 33342 nuclear stain (DNA, blue, fourth row) following treatment with dimethyl sulfoxide (DMSO, left column) or RAP (right column), respectively. Scale bars, 5 µm.

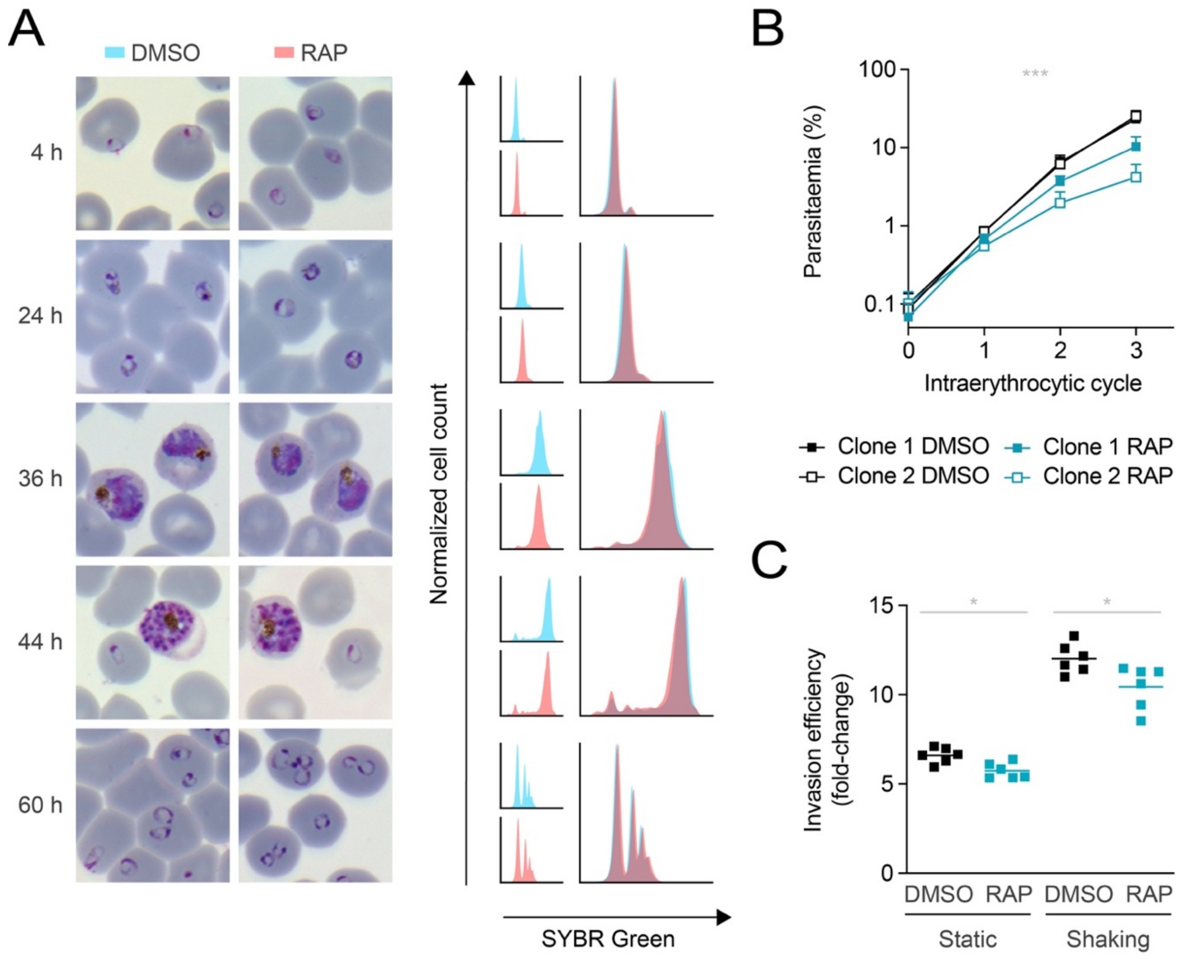

***SI Appendix*, Fig. S5.** Impaired fitness of *in vitro* cultivated *Plasmodium falciparum* in the absence of PV5.

(*A*) *Pf*PV5-deficient parasites mature normally *in vitro*. Tightly synchronized *pv5-3xHA:loxP* ring stages were treated with dimethyl sulfoxide (DMSO, blue) or rapamycin (RAP, red) and visualized by Giemsa staining 4, 24, 36, 44 and 60 hours later (left). In parallel, nuclear SYBR Green fluorescence was quantified by flow cytometry. Individual and merged histograms are depicted (right). Results are representative of two independent experiments.

(*B*) Asexual parasite proliferation is impaired upon loss of *Pf*PV5. Shown are growth curves of two independent *pv5-3xHA:loxP* clonal lines upon treatment with DMSO or RAP, respectively. Averaged parasite multiplication rates are 7.6 (DMSO) and 4.6 (RAP). Shown are mean values +/- SD. ***, $P<0.001$; Two-way ANOVA. N=6 independent infections.

(*C*) Impaired schizont to ring stage transition in the absence of *Pf*PV5. Schizonts from DMSO- and RAP-treated *pv5-3xHA:loxP* cultures were added to fresh erythrocytes and incubated under static or shaking conditions for 24 hours. Shown is the fold-change in parasitaemia, depicted as individual and mean values (bars). *, $P<0.05$; paired *t*-test. N=6 independent infections.

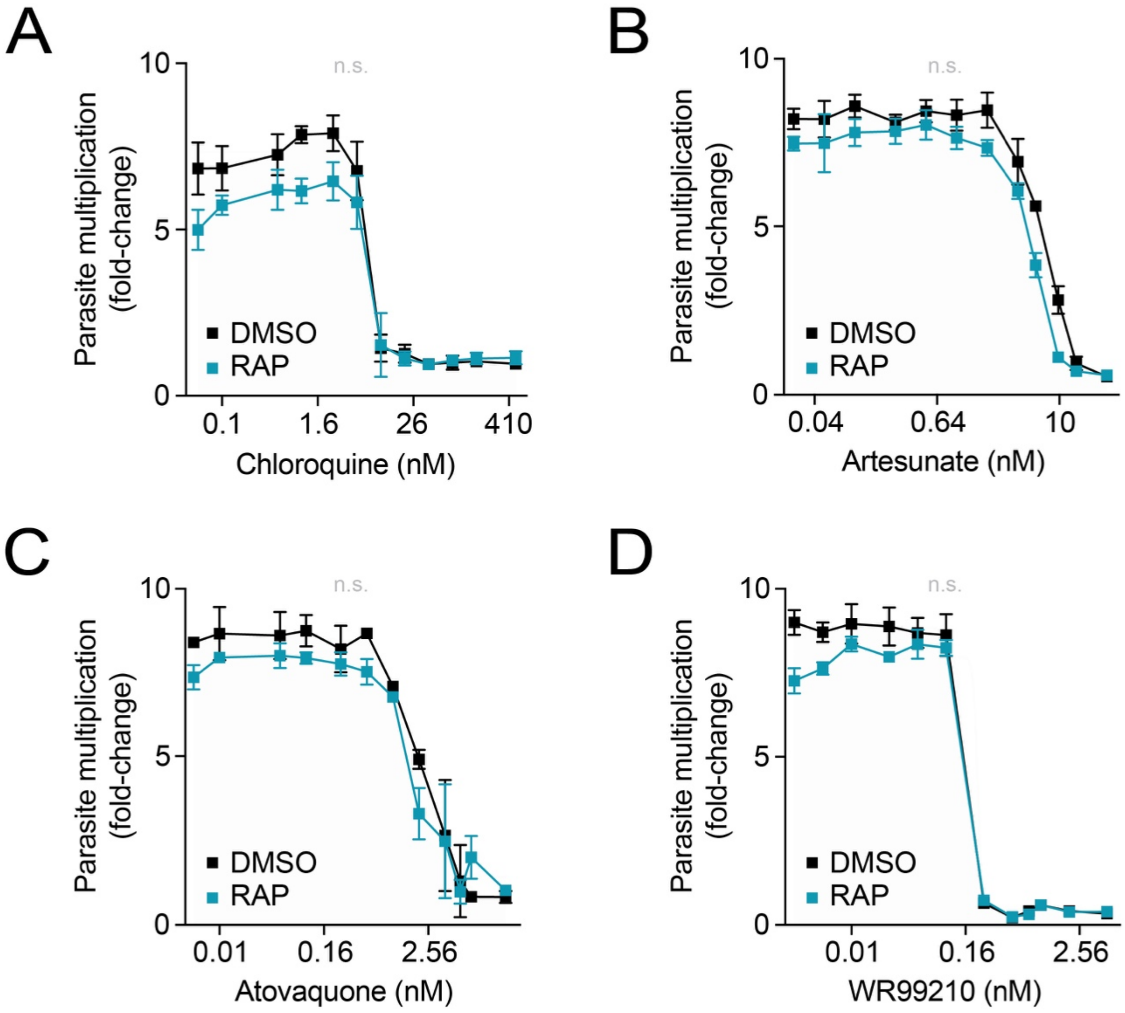

***SI Appendix*, Fig. S6.** Normal drug sensitivity of PV5-deficient *P. falciparum* parasites *in vitro*.

(*A*-*D*) Shown are dose-response analyses of dimethyl sulfoxide (DMSO)- and rapamycin (RAP)-treated *pv5-3xHA:loxP* parasites. Static ring stage cultures were treated with varying concentrations of chloroquine (*A*), artesunate (*B*), atovaquone (*C*) or WR99210 (*D*) in the presence of DMSO or RAP, and the transition into the following intraerythrocytic cycle was quantified. IC_{50} values: chloroquine, 6.9 and 7.8 nM; artesunate, 7.5 and 5.7 nM; atovaquone, 2.2 and 1.6 nM; WR99210, 0.16 and 0.21 nM for DMSO- and RAP-treated parasites, respectively. Depicted are mean values +/- SD. n.s., non-significant; fitting of IC_{50} values following non-linear regression, N=3.

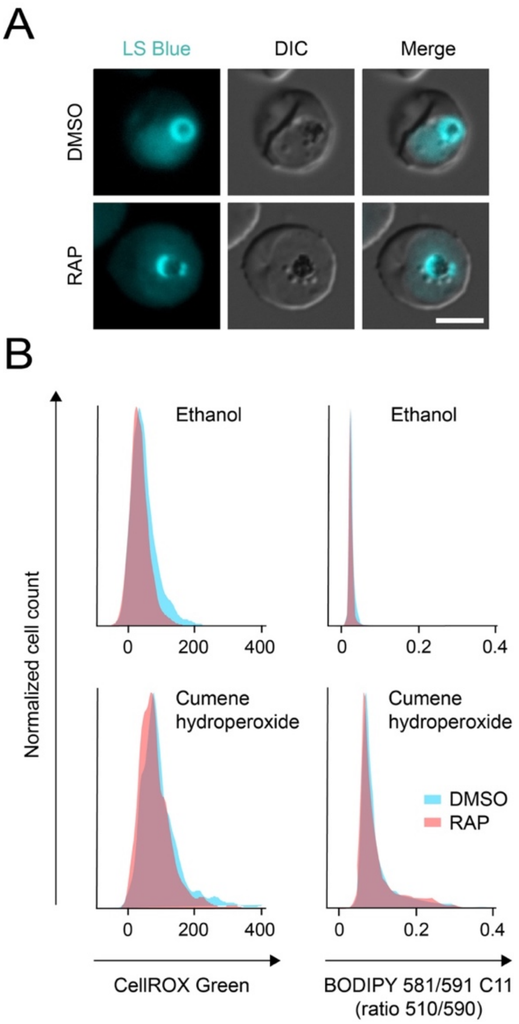

***SI Appendix*, Fig. S7.** Absence of *Pf*PV5 does not cause dissipation of the vacuolar pH gradient nor an increase in oxidative stress.

(*A*) *Pf*PV5-deficient parasites maintain an intact and acidic FV. Synchronized *pv5-3xHA:*loxP parasites were treated with dimethyl sulfoxide (DMSO) or rapamycin (RAP) from ring stage onward and were stained with Lysosensor Blue DND-167 (LS Blue) 36 hours later. Shown are the LS Blue channel (cyan, left), differential interference contrast images (DIC, centre) and a merge (right). Scale bar, 5 µm.

(*B*) No increased oxidative stress in the absence of *Pf*PV5. Synchronized *pv5-3xHA:loxP* parasites were treated with DMSO (blue) or RAP (red) from ring stage onward, stained with the oxidative stress sensor CellROX Green (left) or with the ratiometric lipid peroxidation dye BODIPY 581/591 C11 (right) 36 hours later and analysed by flow cytometry. In addition, parasites had been treated with the oxidative stress-inducing agent cumene hydroperoxide (bottom) or with ethanol as the solvent control (top). Shown are the histograms of CellROX Green fluorescence intensity or of the 510/590 nm fluorescence ratio of BODIPY 581/591 C11. Results are representative of two independent experiments.

***SI Appendix*, Movie S1.** Absence of *Pf*PV5 ablates haemozoin movement within the food vacuole of *Plasmodium falciparum*.

Shown are differential interference contrast recordings of dimethyl sulfoxide (DMSO, left) and rapamycin-treated (RAP, right) *pv5-3xHA:loxP* parasites 36 hours following invasion. The video contains 120 frames shown at a 4x acceleration. Elapsed time is indicated in the upper right corner. Scale bar, 5 μm.

***SI Appendix*, Table S1. Haemozoin crystal morphometry.**

	**WT**	***pv5-3xHA:loxP***		WT *vs.* DMSO	WT *vs.* RAP	DMSO *vs.* RAP
		**DMSO**	**RAP**			
Area (μm^2)	0.164 (±0.088)	0.107 (±0.052)	0.102 (±0.039)	***	***	n.s.
Aspect ratio	3.673 (±1.047)	2.781 (±1.061)	1.604 (±0.337)	***	***	***
Branching (%)	0	27.5	96.1			

n.s., non-significant; ***, P<0.001; One-way ANOVA and Tukey's multiple comparison test. N>300 crystals.

SI Appendix, Table S2. Primer sequences.

Primer Name	Primer Sequence (restriction sites)	WT (bp)[a]	INT (bp)[b]	EX (bp)[c]	Use[d]	Target	Reference
NT-PbPV5-F-BamHI	agttttggatccaaaatgaaattttatagcatttttgcaattg	707			TV	NT Pb*PV5*	This study
NT-PbPV5-R-SacII	agtattccgcggtttaaataacatttctgatttttttctcc				TV	NT Pb*PV5*	This study
5'-PbPTEX88-F-BamHI	aaatatggatcctttttttgtgaaataagttgtttggtg	1,496			TV	5' Pb*PTEX88*	This study
5'-PbPTEX88-R-BamHI	atatatggatccaattttggggatttcaatctttttaag				TV	5' Pb*PTEX88*	This study
5’-PbHSP101-F-BamHI	ttaaaaggatccaaaaattatacaatgcgtgtggc	1,489			TV	5' Pb*HSP101*	This study
5’-PbHSP101-R-BamHI	ttttcaggatccatttatagtaaatatagatataattttatcttcattc				TV	5' Pb*HSP101*	This study
mCherry-F-AatII	aatttagacgtcatgaaggtgagcaagggcg	735			TV	*mCherry*	This study
mCherry-R-AatII	aatttagacgtccttgtacagctcgtccatg				TV	*mCherry*	This study
T7	taatacgactcactataggg		1,206		GT	T7	-
5’-PbPV5-F	gtgggtcgttatttgtattttttaatattagg	1,440			GT	5' Pb*PV5*	This study
CT-PbPV5-R1	gatggatcataaccagcaacg				GT	CT Pb*PV5*	This study
5’-PbPTEX88-F	agtacgaagatataatttgaaaagcc		1,091		GT	5' Pb*PTEX88*	This study
5’-PbHSP101-F	tgcaactacattttattacgccc		1,177		GT	5' Pb*HSP101*	This study
CT-PbPV5-F	taaacccgttgatgaaaacactactgttg	261			qPCR	CT Pb*PV5*	This study
CT-PbPV5-R2	ggatcataaccagcaacgtaaagagc				qPCR	CT Pb*PV5*	This study
Pb18S-F	aagcattaaataaagcgaatacatccttac	134			qPCR	Pb*18S-rRNA*	(1)
Pb18S-R	ggagattggttttgacgtttatgtg				qPCR	Pb*18S-rRNA*	(1)
NT-PfPV5-F	attgattgttattatcatttccag	24			gRNA	NT Pf*PV5*	This study
NT-PfPV5-R1	aaacctggaaatgataataacaat				gRNA	NT Pf*PV5*	This study
5’-PfPV5-F	aatgcgggggaggagaaccc	836	730	2,006 / 1,260	GT	5’ Pf*PV5*	This study
NT-PfPV5-R2	acaactccatcctatcaaaattaaag				GT	NT Pf*PV5*	This study
CT-PfPV5-F	catgatcattatgtctaaatatagaacc	670			GT	CT Pf*PV5*	This study
3’-PfPV5-R	atgtgaaaaaacttacaaactatataccc		561	2,006 / 1,260	GT	3’ Pf*PV5*	This study
LoxP-F	taacttcgtatagcatacattatacg				GT	*loxP*	This study
LoxP-R	aacttcgtataatgtatgctatacg				GT	*loxP*	This study

[a] Sizes of Wild-type-specific PCR products or mCherry.

[b] Sizes of integration-specific PCR products.

[c] Sizes of excision-diagnostic PCR products (non-excised / excised).

[d] Primers used for construction of Transfection Vectors (TV), for GenoTyping (GT), quantitative real-time PCR (qPCR), or for guide RNAs (gRNA). Used primer combinations are indicated in Figure 3*A* and in *SI Appendix*, Figures S1*A* and S2*A*.

SUPPLEMENTARY REFERENCES

1. J. Friesen *et al.*, Natural immunization against malaria: causal prophylaxis with antibiotics. *Sci Transl Med* **2**, 40ra49 (2010).
